# Prevalence, Comorbidities, and Current Management of Chronic Spontaneous Urticaria in Japan: Retrospective Claims Database Study

**DOI:** 10.1111/1346-8138.17943

**Published:** 2025-09-08

**Authors:** Atsushi Fukunaga, Yuko Kishi, Yoshinori Sunaga, Kazuhiko Arima

**Affiliations:** ^1^ Osaka Medical and Pharmaceutical University Takatsuki Osaka Japan; ^2^ Sanofi K.K. Shinjuku Tokyo Japan

**Keywords:** chronic spontaneous urticaria, database, epidemiology, Japanese, real‐world

## Abstract

Chronic spontaneous urticaria (CSU) affects 0.5%–1.0% of the population worldwide. However, information on the prevalence, comorbidities, and treatment patterns of CSU in Japan is limited. This observational study consisted of a cross‐sectional and longitudinal part. The prevalence and incidence of CSU were estimated using the JMDC claims database (JMDC Inc., Tokyo, Japan). CSU was defined as either ‘idiopathic urticaria’, ‘other urticaria’ or ‘unspecified urticaria’ based on ICD‐10 codes and prescribed H_1_‐antihistamines for ≥ 6 weeks over a 3‐month period. Comorbidities, current treatments, and healthcare resource utilization in patients with CSU were also evaluated. The prevalence of CSU increased from 1.2% in 2016 to 1.6% in 2021 with a peak in children, while the incidence of CSU remained constant (0.7%–0.8%). Both prevalence and incidence were higher in females. Comorbid allergic rhinitis and conjunctivitis were frequent in the overall population, while comorbid asthma and atopic dermatitis were frequent in patients aged ≤ 11 years. During the 1‐year observational period, approximately 95% of patients used H_1_‐antihistamines, followed by topical steroids, which are not recommended in the guidelines. Patients receiving the treatment in the same step as the initial month constituted the highest proportion represented during the following year. Oral corticosteroid use was relatively high, including in children, whereas omalizumab use was limited. The estimated recent prevalence and incidence of CSU in Japan were 1.6% and 0.8%, respectively. We also described patient characteristics and healthcare resource use. The present study encourages standardized practice for uncontrolled patients with CSU in Japan.

**Trial Registration:** UMIN Clinical Trials Registry: UMIN‐CTR 000051032

## 1 Introduction

Chronic urticaria, a highly common skin disease, is characterized by pruritic wheals (hives), angioedema, or both for more than 6 weeks [1, 2]. In the Japanese Guidelines for Diagnosis and Treatment of Urticaria 2018 [3], chronic urticaria refers to chronic spontaneous urticaria (CSU) whereas, in the international guidelines [2], it refers to both CSU and chronic inducible urticaria (CIndU). CSU, in which wheals spontaneously appear for 6 weeks or more without an obvious trigger, is the most common urticaria type. A cross‐sectional observational survey conducted in primary care institutes in a specific area of Japan found that CSU comprised approximately 62% of urticaria subtypes [4], and it is thought that 0.5%–1.0% of the population worldwide is affected by CSU at any time [2]. In Japan, a recent analysis of real‐world data using data from the 2019 National Health and Wellness Survey and 2018 international census projections estimated a weighted 12‐month prevalence of CSU of 1.1%, of whom 62.3% were female [5].

CSU and other forms of chronic urticaria have a large impact on quality of life and functioning, especially when not managed optimally [1, 6, 7]. A cross‐sectional, non‐interventional, observational, web‐based survey conducted in Japan found that chronic urticaria patients with Urticaria Control Test (UCT) scores of < 12 had higher work productivity loss and activity impairment versus patients with UCT scores of ≥ 12 [6]. Further, many patients reported high levels of dissatisfaction with their health state (particularly during periods of severe disease) and with current treatment [6]. Similarly, a more recent web‐based cross‐sectional survey in Japan found that, based on UCT scores, two‐thirds of patients had poor or insufficient disease control and such patients had worse health‐related quality of life outcomes [7]. These results are supported by other studies on the impact of CSU on quality of life, especially in patients with more severe disease, which have documented wide‐ranging negative impacts on mobility, sleep, mental status, and energy. This impact on QoL has wider consequences on work, relationships, and social life [8, 9]. One comparative survey found that patients with CSU with incomplete treatment responses had significant reductions in quality of life, comparable to those of patients with other chronic inflammatory diseases, such as rheumatoid arthritis or insulin‐dependent diabetes [10].

Management of urticaria follows a stepwise approach, with initial use of second‐generation, non‐sedating H_1_‐antihistamines being generally effective. However, H_1_‐antihistamines are ineffective in some patients, even with dose up‐titration [1, 2]. Patients who are resistant to up‐dosing of H_1_‐antihistamines or concomitant use of multiple antihistamines/antileukotrienes have few treatment options. Omalizumab (anti‐IgE monoclonal antibody) was approved in Japan in March 2017 for CSU poorly responsive to conventional therapy and has provided greater opportunity to manage refractory CSU [11]. The real‐world safety and effectiveness of omalizumab in Japanese patients has been confirmed in a 52‐week, open‐label, single‐arm, observational post‐marketing study [11]. Dupilumab (anti‐interleukin‐4 receptor monoclonal antibody) was approved for CSU in Japan in February 2024 and is not included in this study.

On this background and rationale, this healthcare insurance database study seeks to provide epidemiological data related to the prevalence, comorbidities, and treatment patterns of patients with CSU in Japan.

## 2 Methods

### 2.1 Study Design and Settings

Annual prevalence and incidence of CSU between 2016 and 2021 were analyzed for each analysis year (i.e., January to December) using a cross‐sectional analysis approach.

Comorbidities, current treatments, and healthcare resource utilization in patients with CSU were evaluated using a longitudinal analysis approach. The index period to identify the eligible patients was from January 2016 to December 2020, with the index month being the earliest month of meeting eligibility criteria during the index period. The data period to extract data used for the analysis was from January 2005 to December 2021, which was divided into a baseline period of 12 months before, and including, the index month and a follow‐up period of at least 12 months from, and following, the index month. As omalizumab approval in Japan was in March 2017, for the analysis of omalizumab use, the index period was from March 2017 to December 2020, with the index date being the earliest omalizumab prescription date, and the data period was from March 2016 to December 2021.

### 2.2 Data Source

Data for this study was extracted from the JMDC claims database (JMDC Inc., Tokyo, Japan), a nationwide, anonymized database that has accumulated claims data from a range of health insurance sources [12]. The cumulative dataset, which contains medical and pharmacy claims data for approximately 13 million insured Japanese subjects (as of April 2022). This study used the Payer database category, which consists of a ledger of enrollments belonging to health insurance associations, all claims issued when the subscriber visits a medical institution, and annual health checkup results.

### 2.3 Definition of Patients With CSU

This study defined patients with CSU as those (i) diagnosed with the following urticaria ICD‐10 codes: L50.1 [idiopathic urticaria], L50.8 [other urticaria], L50.9 [urticaria, unspecified], and (ii) prescribed H_1_‐antihistamines for more than 6 weeks within a 3‐month period. This study defined patients with new‐onset CSU as (i) those who met the CSU definition above, and (ii) without a diagnosis of CSU for at least 1 year before meeting the CSU definition.

### 2.4 Calculation for CSU Prevalence and Incidence

**FIGURE 1:**
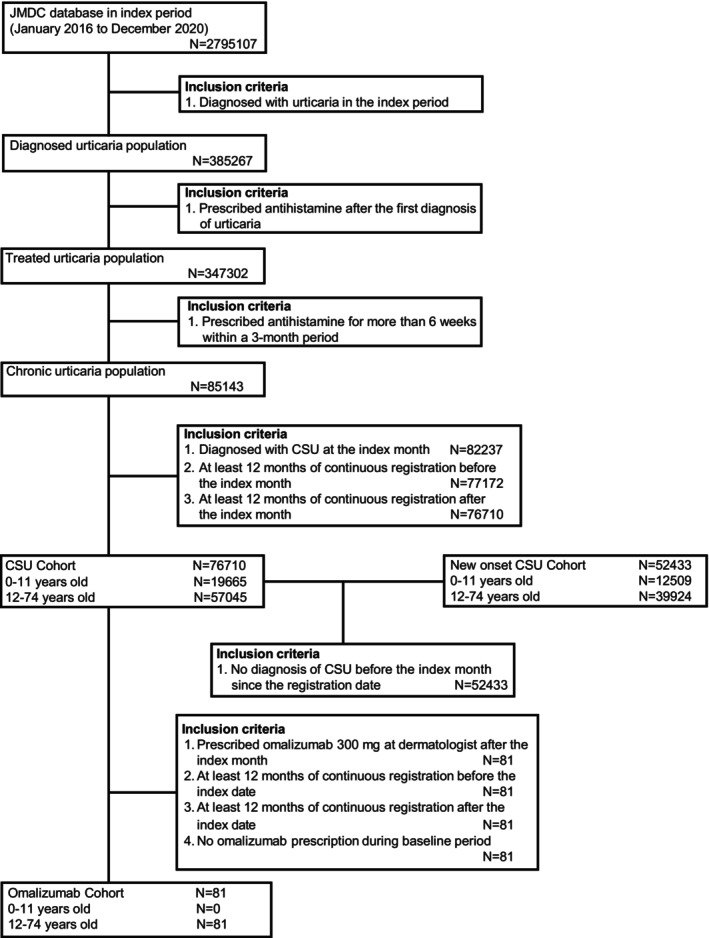
Patient flow for longitudinal analysis.

To estimate the prevalence of CSU, we used a cross‐sectional cohort consisting of patients enrolled continuously in the JMDC database during each analysis year (2 795 107 patients; 2016–2021) as a denominator and calculated the percentage of CSU patients (Figure 1). To estimate the incidence of CSU, we used the abovementioned cohort but excluded those diagnosed with CSU in the previous year as a denominator and calculated the percentage of new‐onset CSU patients. Age was categorized by decade groupings (for ages 0–9 years to 60–69 years) and for ages ≥ 70 years, whereas sex was categorized as male or female. Prevalence was estimated using the JMDC population, while estimates of standardized prevalence results adjusted for the Japanese population were calculated based on the JMDC population.

### 2.5 Definition of Longitudinal Analysis Cohort

For analysis of comorbidities, treatment patterns, CSU control, and healthcare resource utilization (HCRU), we used a longitudinal cohort consisting of patients enrolled continuously in the JMDC database in index period (January 2016 to December 2020) (Figure 1). The month that patients met the CSU definition was defined as the index month. The CSU Cohort included patients with CSU who had been registered continuously at least 12 months before and including the index month (baseline period) and at least 12 months after the index month (follow‐up period). The New‐onset CSU Cohort (herein ‘NeO CSU Cohort’) included patients from the CSU Cohort but with no diagnosis of CSU before the index period since database registration. The Omalizumab Cohort included patients from the CSU Cohort but with the additional requirements of (i) having been prescribed omalizumab 300 mg by a dermatologist after meeting the CSU definition (first omalizumab prescription date was defined as index date), (ii) at least 12 months of continuous registration before and including the index date (baseline period) with no omalizumab prescription during the baseline period, (iii) at least 12 months of continuous registration after the index date (follow‐up period).

### 2.6 Outcomes for Longitudinal Cohort Analysis

Comorbidities in the CSU Cohort and NeO CSU Cohort were those observed at least once during the baseline period and were classified according to pertinent ICD‐10 diagnosis code (Table S1). Treatment patterns included the following variables observed in the follow‐up period: (i) prescription type of drugs recommended in the Japanese Treatment Guidelines [3] (see Table S2 for details); (ii) prescribed doses of H_1_‐antihistamines, as well as topical corticosteroids (TCS) and oral corticosteroids (OCS); (iii) treatment intensity in terms of guideline‐based step level as follows: Step 1; dose of H_1_‐antihistamines (this study subdivided Step 1 into Step 1a; standard dose, Step 1b; up‐dosing or combination), Step 2; dose of H_2_‐antihistamines, LTRA, as part of options within Step 2 of the Japanese Treatment Guidelines [3], and Step 3; dose of omalizumab, cyclosporine, or OCS at index and at 2 to 3, 4 to 6, and 7 to 12 months during the follow‐up period; (iv) cumulative dose per year of TCS and OCS as well as omalizumab. CSU control in the follow‐up period was described in terms of proportion and duration of long‐term maintenance of Step 1a H_1_‐antihistamine treatment, defined as more than 56 days of prescription duration. HCRU included the following variables observed in the follow‐up period: (i) outpatient visits (sum of medical care days in outpatient claims); (ii) number and proportion of hospitalized patients with or without CSU and duration of hospitalization as inpatient or undergoing diagnosis/procedure combination; (iii) clinical laboratory tests (number and proportion); (iv) prescription count for medications related to CSU; (v) prescription count for medications unrelated to CSU. Persistence of omalizumab treatment in terms of the time to treatment discontinuation of omalizumab with no dose within 84 days of the last being assumed to be discontinuation (treatment persistence at 6 and 12 months, median days to treatment discontinuation) and the number of patients restarting omalizumab and time to restart were described in the Omalizumab Cohort.

### 2.7 Statistical and Ethical Considerations

Since this was an observational study based on claims data only, formal sample size was not calculated and all patients who met the inclusion criteria were included in the analysis.

Descriptive statistical methods were used with categorical variables summarized as counts (n) and proportions (%) with two‐sided exact (Clopper‐Pearson) 95% confidence interval (CI). Continuous variables were also summarized with descriptive statistics, including number, mean, median, standard deviation (SD), quartiles (Q1 and Q3), interquartile range (IQR), minimum, and maximum. Time to event variables were summarized with Kaplan–Meier methods, including the event‐free probability and 95% CI at specific time points.

Data analytics were performed using Amazon Redshift (Amazon.com Inc.) while SAS version 9.4 (SAS Institute Inc., Cary, NC, USA) was used for all statistical analyses.

Although this study used anonymized data and did not collect, use, or transmit individually identifiable patient data, the study sponsor submitted the study protocol to the Institutional Review Board for review and approval. The study was registered in the UMIN Clinical Trials Registry (UMIN‐CTR 000051032).

## 3 Results

**FIGURE 2:**
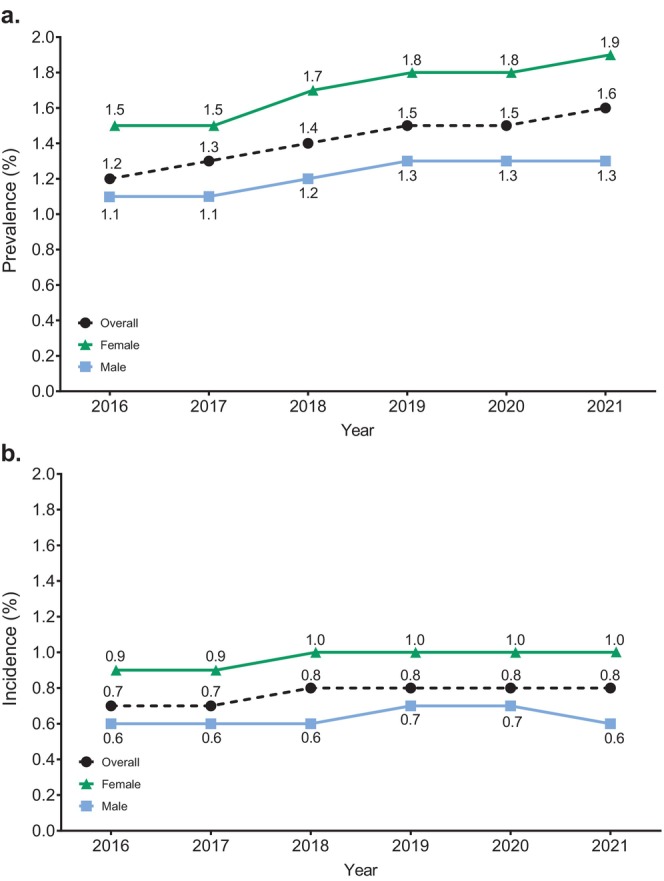
(a) Prevalence and (b) Incidence of CSU from 2016 to 2021.

**FIGURE 3:**
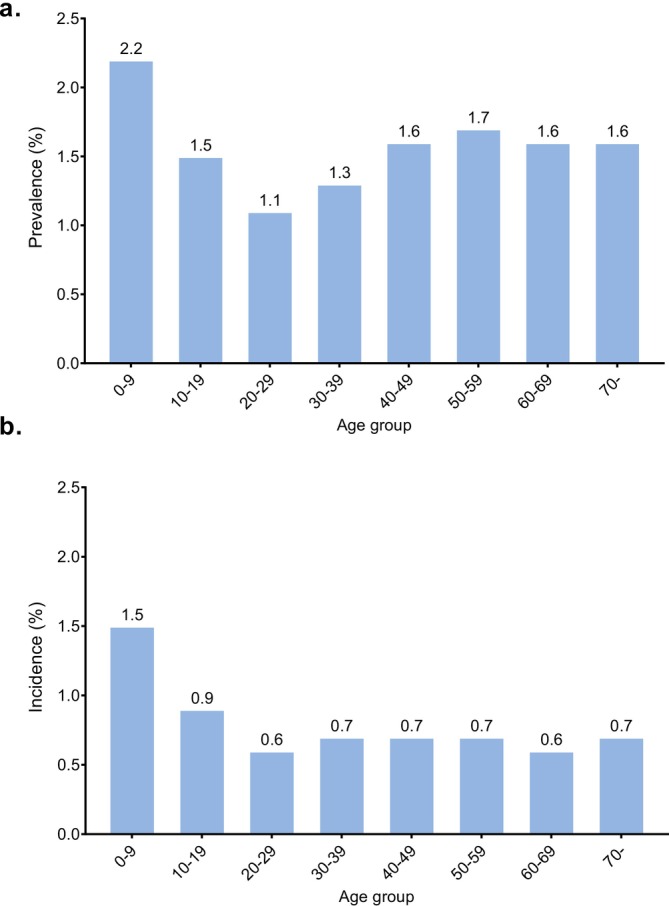
(a) Prevalence and (b) Incidence of CSU by age group in 2021.

According to ICD‐10 codes, the following number (percentage of age group) of children and adolescents/adults, respectively, were included in the CSU Cohort: L50.1 (idiopathic urticaria) – 103 (0.5%) and 196 (0.3%); L50.8 (other urticaria) – 808 (4.1%) and 8116 (14.2%); L50.9 (urticaria, unspecified) – 18 955 (96.4%) and 49 905 (87.5%). Almost all patients classified under L50.8 had chronic urticaria (99.5% for both age groups) while other types of urticaria within L50.8 were extremely rare (< 1%) (Table S3). Prevalence (Figure 2a) and standardized prevalence (Table S4) of CSU were determined annually from 2016 to 2021, and they were found to gradually increase; the prevalence and the standardized prevalence rose from 1.2% in 2016 to 1.6% in 2021, and the standardized prevalence rose from 1.3% to 1.6% over this period. On the other hand, incidence (new‐onset CSU cases) was relatively stable during this period (Figure 2b, Table S5). The prevalence and incidence in females were higher than in males during this period. The prevalence of CSU was highest in children less than 10 years old (2.2%) and lowest in the twenties (1.1%) among all generations. The incidence of CSU was also highest in children (Figure 3).

**FIGURE 4:**
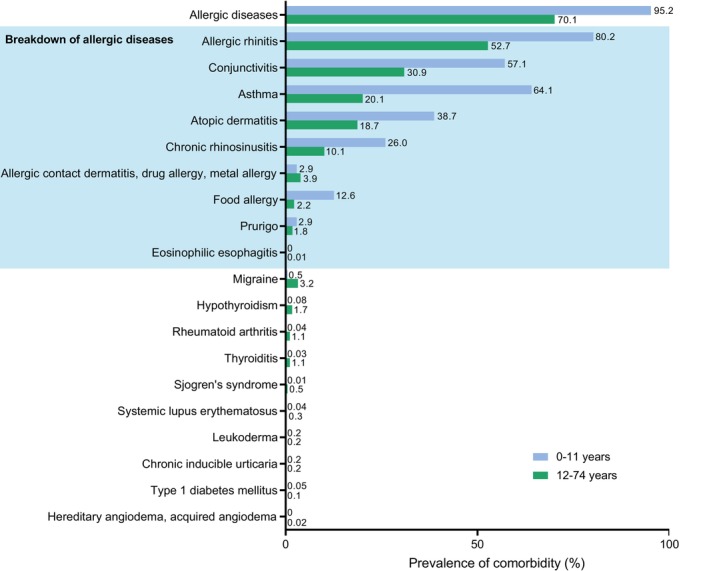
Common comorbid conditions in the CSU Cohort in children (0–11 years) and adolescents/adults (12–74 years) during the baseline period.

To describe comorbidities in the patients with CSU, we separately analyzed them between patients aged ≤ 11 years (children) and ≥ 12 years (adolescents/adults) because patient management options for these two populations are different [13]. Demographics for the CSU and NeO CSU Cohorts (Table 1) show that the median age at index was 6.0 years and 5.0 years, respectively, for children and 42.0 and 51.0 years, respectively, for adolescents/adults; further, the ratio of males to females was higher than unity in children (CSU, 1.21; NeO CSU, 1.19) and lower in adolescents/adults (CSU, 0.88; NeO CSU, 0.89). Comorbid allergic disease was observed in 95.2% of children and in 70.1% of adolescents/adults (Figure 4). Most of the comorbid allergic diseases were more common in children. Allergic rhinitis, asthma, and conjunctivitis were particularly prevalent among children, occurring in 80.2%, 64.1%, and 57.1%, respectively. Of note, food allergy was relatively common in children compared with adolescents/adults (12.6% vs. 2.2%).

**TABLE 1:** Demographics for CSU and NeO CSU Cohorts.

	CSU Cohort		NeO CSU Cohort	
	0–11 years (*N* = 19 665)	12–74 years (*N* = 57 045)	0–11 years (*N* = 12 509)	12–74 years (*N* = 39 924)
Age at index month, years				
Mean (SD)	5.9 (3.0)	40.1 (14.3)	5.7 (3.0)	40.1 (14.4)
Median (1Q, 3Q)	6.0 (4.0, 8.0)	42.0 (31.0)	5.0 (3.0, 8.0)	51.0 (31.0, 51.0)
Age category, *n* (%)				
12–19		8468 (14.8)		5770 (14.5)
20–29		4236 (7.4)		3163 (7.9)
30–39		11 214 (19.7)		7998 (20.0)
40–49		17 049 (29.9)		11 728 (29.4)
50–59		12 578 (22.0)		8613 (21.6)
60–69		3220 (5.6)		2421 (6.1)
≥ 70		280 (0.5)		231 (0.6)
Gender, *n* (%)				
Male	10 760 (54.7)	26 691 (46.8)	6788 (54.3)	18 786 (47.1)
Female	8905 (45.3)	30 354 (53.2)	5721 (45.7)	21 138 (52.9)

Abbreviations: NeO, New‐onset; Q1, Q3, interquartile range; SD, standard deviation.

**FIGURE 5:**
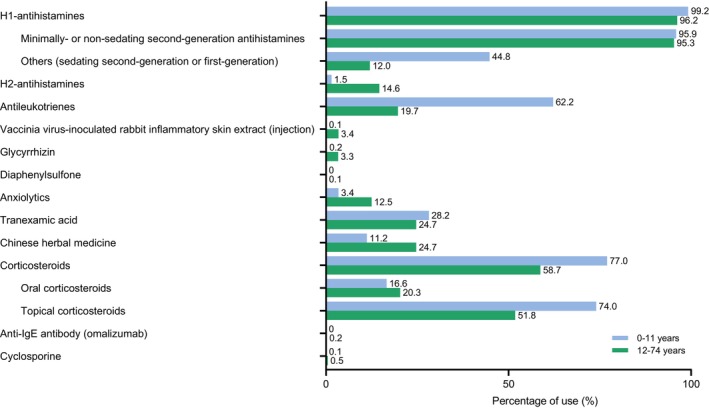
Medications prescribed in CSU Cohort in children (0–11 years) and adolescents/adults (12–74 years) during the follow‐up period.

Regarding medications used in CSU patients, minimally or non‐sedating second‐generation H_1_‐antihistamines (children 95.9%; adolescents/adults 95.3%) were predominantly used, followed by TCS (children 74.0%; adolescents/adults 51.8%), tranexamic acid (children 28.2%; adolescents/adults 24.7%), Chinese herbal medicine (Kampo, children 11.2%; adolescents/adults 24.7%), and antileukotrienes (children 62.2%; adolescents/adults 19.7%) (Figure 5). Compared with older patients, children received a higher proportion of TCS, antileukotrienes, sedating second‐generation or first‐generation H_1_‐antihistamines, and tranexamic acid. On the other hand, Chinese herbal medicine, OCS, H_2_‐antihistamines, and anxiolytics were prescribed to a higher proportion of adolescents/adults compared with children. OCS described as a Step 3 medication in the Japanese Treatment Guidelines [3] was used 16.6% in children and 20.3% in adolescents/adults. Other Step 2 medications such as vaccinia virus–inoculated rabbit inflammatory skin extract, glycyrrhizin, and diaphenylsulfone, and Step 3 medications cyclosporine and omalizumab were prescribed infrequently. Omalizumab was prescribed in 101 (0.2%) patients aged 12 years and older, consistent with labeled age.

The primary treatment goal of CSU is to achieve a symptom‐free condition by continuous use of medications such as H_1_‐antihistamines as Step 1. Children and adolescents/adults received H_1_‐antihistamines regardless of other treatments for a median (Q1, Q3) duration of 170.6 (91.1, 276.2) days/year and 159.7 (71.0, 292.4) days/year, respectively (Table S6). The results of long‐term maintenance in Step 1a show that approximately one‐third of CSU patients were able to be maintained long‐term with Step 1a level treatment (standard dose of single H_1_‐antihistamine only) at a median (Q1, Q3) prescription duration of 119.0 (76.0, 229.0) days (Table S6). Further, among adolescents/adults, 6.5% and 17.1% of patients received combination prescriptions and double dose prescriptions of H_1_‐antihistamines (i.e., Step 1b in this study), respectively, which are recommended as Step 1 treatment in the Japanese Treatment Guidelines [3] (13.3% and 14.1%, respectively, in children).

Regarding OCS use, the median cumulative doses of OCS were 80.1 mg/year (prednisolone equivalents) in adolescents/adults and 26.7 mg/year in children, with the median (Q1, Q3) prescription duration being 14.0 [5.0, 58.0] days and 5.0 [2.0, 14.0] days/year, respectively (Table S7).

**FIGURE 6:**
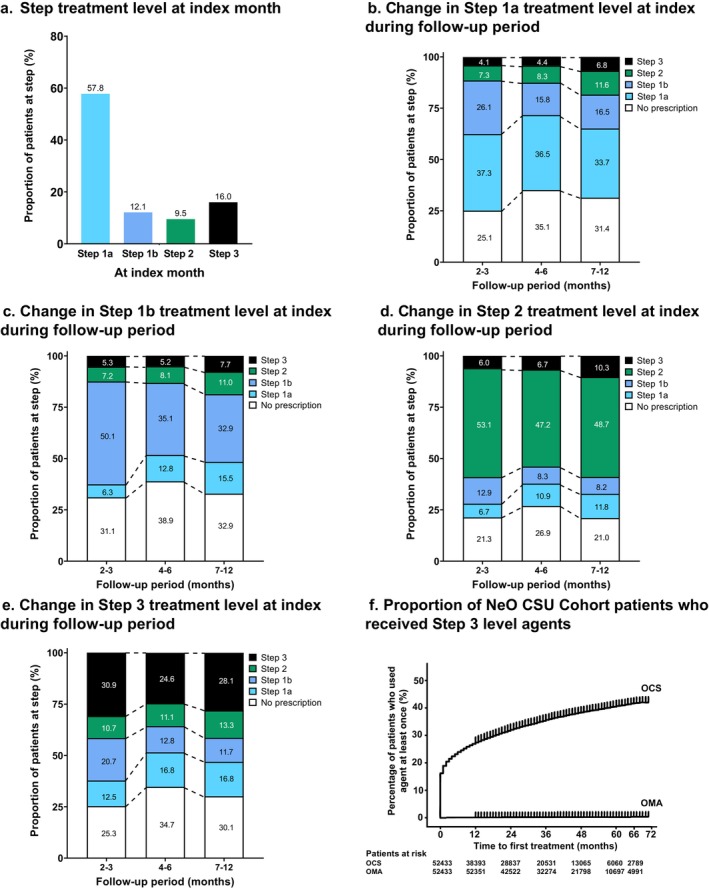
(a–e) The proportions of CSU patients (CSU Cohort) aged 12–74 years in each treatment intensity level step at the index month and during the follow‐up period by treatment intensity level at the index month and (f) Proportion of NeO CSU Cohort patients who received Step 3 level agents.

The proportions of CSU patients aged 12–74 years in each treatment intensity step level at the index month and during the follow‐up period are shown in Figure 6a–e. At the index month, approximately 58%, 12%, 10%, and 16% of adolescent/adult patients received Step 1a, Step 1b, Step 2, and Step 3 level treatment, respectively. At each follow‐up period, the greatest proportion of patients in each treatment intensity step was the same as at the index month. Patients who received Step 2 at index appeared to be the least likely to transition to other treatment intensity levels in subsequent follow‐up periods, with the proportion of patients receiving Step 2 treatment remaining at approximately 50%. The proportion of patients initially at Step 3 who remained at Step 3 decreased to 30.9% at 2–3 months, and no further big decrease was observed for 1 year. To provide further insights into the trajectory of the ‘no prescription’ population that appeared during the follow‐up period, we created a Sankey diagram of adolescent/adult patient migration at each transition step (Figure S1). This highlighted that approximately one third of the ‘no prescription’ population returned to treatment at 4–6 months and 7–12 months.

The proportion of patients who first received Step 3 level medications in the NeO CSU Cohort is shown in Figure 6f. This shows that approximately 16% of patients received OCS immediately after new onset CSU and approximately 40% of patients received OCS during observed periods up to 6 years. In this cohort, omalizumab was rarely used.

HCRU in terms of outpatient visits, hospitalizations, laboratory tests, and prescriptions in CSU patients is shown in Table 2. CSU‐related outpatient visits comprised a median (Q1, Q3) of 4.0 (2.0, 8.0) and 5.0 (2.0, 9.0) days per year in children and adolescents/adults, respectively. CSU‐related hospitalizations comprised a median of 2.0 (1.0, 6.0) and 8.0 (4.0, 19.0) days per year in children and adolescents/adults, respectively. Clinical laboratory tests were not widely performed for CSU patients; blood and biochemical tests were performed in only < 10% of children and < 15% of adolescent/adult CSU populations. Coagulation tests were used in < 1% of CSU patients. Intracutaneous tests were far less frequently performed. CSU‐associated prescriptions were also high, with a median of 11.0 (7.0, 18.0) times/year in children and 7.0 (4.0, 11.0) times/year in adolescents and adults.

**TABLE 2:** Healthcare resource utilization (CSU Cohort).

	0–11 years (*N* = 19 665)		12–74 years (*N* = 57 045)	
Outpatient visits (All‐cause), days/year				
Mean (SD)/Median (Q1, Q3)	6.4 (6.9)	4.0 (2.0, 8.0)	6.5 (6.6)	5.0 (2.0, 9.0)
Outpatient visits (CSU‐related), days/year				
Mean (SD)/Median (Q1, Q3)	6.4 (6.9)	4.0 (2.0, 8.0)	6.5 (6.6)	5.0 (2.0, 9.0)
Hospitalizations (All‐cause), days/year				
*n* (%)	1144 (5.8)		3025 (5.3)	
Mean (SD)/Median (Q1, Q3)	8.1 (22.3)	4.0 (2.0, 7.0)	13.7 (26.7)	6.0 (3.0, 12.0)
Hospitalizations (CSU‐related), days/year				
*n* (%)	147 (0.7)		289 (0.5)	
Mean (SD)/Median (Q1, Q3)	9.3 (28.8)	2.0 (1.0, 6.0)	21.6 (39.5)	8.0 (4.0, 19.0)
Clinical laboratory tests (times/year)				
Blood test				
*n* (%)	1917 (9.7)		8449 (14.8)	
Mean (SD)/Median (Q1, Q3)	1.3 (1.1)	1.0 (1.0, 1.0)	2.3 (3.7)	1.0 (1.0, 2.0)
Biochemical test				
*n* (%)	886 (4.5)		7082 (12.4)	
Mean (SD)/Median (Q1, Q3)	1.3 (1.3)	1.0 (1.0, 1.0)	2.7 (4.3)	1.0 (1.0, 3.0)
Coagulation test				
*n* (%)	53 (0.3)		577 (1.0)	
Mean (SD)/Median (Q1, Q3)	2.0 (2.5)	1.0 (1.0, 2.0)	2.6 (4.7)	1.0 (1.0, 2.0)
Intracutaneous test				
*n* (%)	92 (0.5)		133 (0.2)	
Mean (SD)/Median (Q1, Q3)	1.2 (0.4)	1.0 (1.0, 1.0)	1.1 (0.5)	1.0 (1.0, 1.0)
Prescription for CSU‐associated medications (times/year)				
Mean (SD)/Median (Q1, Q3)	13.6 (9.8)	11.0 (7.0, 18.0)	8.7 (8.0)	7.0 (4.0, 11.0)
Prescription for non‐CSU‐associated medications (times/year)				
Mean (SD)/Median (Q1, Q3)	17.7 (13.6)	15.0 (9.0, 23.0)	11.0 (12.0)	8.0 (4.0, 14.0)

Abbreviations: Q1, Q3, interquartile range; SD, standard deviation.

Finally, we exploratorily analyzed the treatment pattern of omalizumab use in patients with CSU. Among the 81 patients assessed for omalizumab persistence, 42.0% remained on omalizumab at 6 months and 16.0% at 12 months, with no dose for 84 days (approximately 3 months) from the last dosing being assumed to be discontinued. In total, 19 patients (23.5%) restarted omalizumab therapy, and the median time to restart was 112 days (Table S8).

## 4 Discussion

This cross‐sectional and longitudinal cohort study based on the JMDC claims database provides several insights into the epidemiology, comorbidities, and treatment patterns of patients with CSU in Japan. In relation to the prevalence and incidence of CSU in Japan, peak prevalence occurred in children (2.2%), and the lower prevalence in young adults (1.1%–1.3%) was consistent with the point prevalence noted in Asia (1.4%) from a systemic review and meta‐analysis [14]. The results of the present study are also supported by those of a recently published questionnaire‐type analysis of real‐world data in Japan that confirmed a similar 12‐month prevalence rate (1.1%) [5]. However, the present study is distinct and novel in several respects from this questionnaire‐type analysis, including in terms of the methodology that used a commercial panel targeting the general population. A cross‐sectional analysis using a national health insurance database in South Korea also reported a point prevalence of 1.4% [15]. Prevalence of CSU tends to be lower in Western countries with a physician‐based online survey conducted in five European countries (United Kingdom, Germany, Italy, France, and Spain) reporting a CSU point prevalence of 0.75% among children [16]. Among adults in the US, a cross‐sectional analysis with electronic health records data reported an overall chronic urticaria rate of 0.23% with a twice higher prevalence rate among females [17]. Another study using the US HealthVerity claims database recently reported an estimated cumulative prevalence of diagnosed CSU of 0.57% (women: 0.80%; men: 0.32%) and the average annual incidence rate was 0.08% [18]. A recent review article supports these findings, highlighting that the point prevalence of CSU tends to be higher in Asian countries (up to 1.4%–2.7%) compared with EU countries and the US (up to 0.11%–0.86%) [19]. Although the reason for these point prevalence differences is unknown, they may include genetic factors, environment, health access and utilization, lifestyle and dietary habits, and cultural factors. The current study also confirms that the peak prevalence occurs in children and that CSU is more common among women.

Prevalence of CSU increased by about 0.1% per year compared with a relatively stable incidence of 0.7%–0.8% across the assessment period for the overall population. These findings imply that the average duration of disease was relatively short (less than 2 years), and a high proportion of new cases resolved within a year. This is consistent with the findings from a population‐based study among adults in Spain, which found that approximately 80% of patients with chronic urticaria were symptom‐free after 1 year [20]. However, the total number of cases was larger than the number of people who underwent disease resolution, and the number of people affected for several years appeared to have accumulated, which is reflected in the increasing prevalence rate over time (Figure 2). This was also shown in the abovementioned study, with around 11% of cases lasting for more than 5 years [20]. Disease duration appeared to be shorter in children than in adolescents or adults, such that the prevalence to incidence ratio among children 0–9 years was approximately 1.5, and many cases in children appeared to resolve within a short term (Figure 3). On the other hand, in elderly patients (≥ 10 years), the ratio of the prevalence to incidence was approximately 2.5, indicative of longer disease durations and suggesting that it is more difficult to treat CSU in this population. Determination of the prevalence of CIndU was out of the scope of this study; however, we observed that the prevalence of CIndU was considerably lower than that of CSU. Definition and data of CIndU are shown in Table S9. This observation is consistent with the recent findings of a questionnaire‐based study in Japan conducted as a cross‐sectional multicentre survey in nine primary dermatology and allergology clinics [4]. We believe the definitions of patients with CSU and CIndU in this present study may reflect diagnostic accuracy to some extent in Japan. Specifically regarding CIndU, another difficulty may be that many doctors may not describe the exact name of the disease when they submit for medical reimbursement.

Regarding comorbidities in patients with CSU, there was a high rate of comorbid allergic diseases in both children (95.2%) and adolescents/adults (70.1%), particularly allergic rhinitis, conjunctivitis, asthma, and atopic dermatitis. This resonated with the fact that most patients with CSU present with features of the type I (autoallergic) endotype, irrespective of the involvement of the type IIb (autoimmune) endotype [21]. In the type I endotype, IgE and Th2 cells, the hallmarks of type II immunity, play a role in the pathogenesis of CSU [22, 23]. Similar to the results of the present study, a previous Scandinavian real‐world study reported that atopic comorbidities were the most frequent, including asthma (19.6%), allergic rhinitis (16.5%), and food allergy (8.2%) [24]. Further, among CSU patients in a large Korean national health insurance database study, allergic rhinitis and asthma were noted to overlap with CSU in 74% and 34% of patients, respectively [15]. Among children, atopic dermatitis is reported to be a risk factor for subsequent CSU development in an analysis of retrospective data from a tertiary pediatric hospital in Greece, describing that children with an early diagnosis of atopic dermatitis were at increased risk for developing CSU (odds ratio 2.9; *p* < 0.001) [25]. Similarly, a recent systematic review related to children with CSU aged less than 12 years reported that, in pediatric CSU, allergic diseases occurred in 28.1% of the population (asthma [15.4%], allergic rhinitis [13.8%], and atopic dermatitis [9.4%]) [26]. These rates are substantially lower than those seen in the current study. This difference might be explained by the difference in cohort nature; patients analyzed for comorbid conditions in the present study must have received H_1_‐antihistamines for 6 weeks or more, which can be used for allergic conditions other than CSU. However, it was also the case that urticaria continued to be registered as a disease in the database.

In the present study, almost all patients received standard formulations and doses of H_1_‐antihistamines (Figure 5). High rates of second‐generation H_1_‐antihistamine use have been noted in an international, observational study of inadequately controlled CSU patients [27], in which 91% of patients received at least one second‐generation H_1_‐antihistamine. Antileukotrienes were prescribed in approximately 60% of children and 20% of adolescents/adults (Figure 5). The rate of antileukotriene use by adolescents/adults was similar to that reported in the international observational study [27], which only includes adult patients. The high rate of antileukotriene use by children might be associated with a high rate of comorbid asthma, as antileukotrienes are recommended from treatment Step 1 as a safe base drug [28]. Whether the prescription of antileukotrienes was truly for CSU or for asthma was unclear with the present study data source. This may be because antileukotrienes have insurance indications for rhinitis and asthma, which are common complications in children with CSU, and are recommended as Step 2 in the current Japanese Treatment Guidelines [3], which differ from international guidelines [2]. Similarly, rates of TCS use in children and adolescents/adults were unexpectedly high, given TCS is not recommended in the CSU treatment guidelines. The rate of OCS use was similar to that reported in the international, observational study (19.9%) [27]. Although OCS use was common in children, and the rate of initial OCS use was similar to that in adolescents/adults, its use in most children was restricted to a median of 5 days per year and around three‐quarters of usage was within a 2‐week period (Table S7). This is in accordance with the Japanese Treatment Guidelines that recommend avoidance of OCS, except for short‐term management of acute exacerbations [3]. On the other hand, use of OCS in adolescents/adults occurred for a longer duration (median 14 days) and at higher cumulative doses than in children (Table S7) with approximately 40% of patients receiving OCS at least once during the observation period of up to maximum of 6 years. This may suggest the need for alternative options to existing Step 3 treatments for CSU patients that can be used for long‐term, in concert with international consensus not recommending the long‐term use of systemic corticosteroids as add‐on treatment [2]. Both TCS and OCS use may relate to treatment of other conditions, such as allergic rhinitis and asthma. In this regard, there is a limitation that prescription data often lack information about medical departments and, in this situation, hospitals with multiple medical departments usually declare internal medicine as their primary medical department. To provide some insight into this, we looked at the breakdown of medical departments of CSU Cohort patients at the index month by imputing lacking department information (approximately 90% in general practitioners and 10% in hospitals) with their primary medical departments. In cases where patients had prescriptions from multiple medical institutes at the index month, they were counted multiple times resulting in the total percentage exceeding 100%. The additional analysis showed the following department breakdown: 60.4% dermatology, 26.6% general internal medicine, and 3.5% pediatrics for patients aged 12 to 74 years; 43.3% pediatrics, 35.6% dermatology, and 18.6% general internal medicine for patients aged 0 to 11 years; all other departments such as allergy, otolaryngology, and respiratory comprised approximately 2% or less each for both populations (Table S10). Although the percentages differed without imputation, the ranking of the top three departments within each age group remained consistent. According to the data with imputation, the majority of index month visits were at practitioner clinics rather than hospitals across both age groups, highlighting the central role of practitioner clinics in CSU management in Japan (Table S11). Furthermore, the breakdown of medical departments where CSU Cohort patients received OCS during the follow‐up period of 1 year was as follows: 52.8% dermatology, 44.2% internal medicine, 21.9% otolaryngology, and 3.2% pediatrics for patients aged 12 to 74 years; 53.1% pediatrics, 50.5% dermatology, 35.4% internal medicine, and 23.0% otolaryngology for patients aged 0 to 11 years; and all other departments such as allergy and respiratory were about 3% or less for both populations (Table S12). Most TCS were prescribed at dermatology departments (69.5% in 12–74 years, 61.5% in 0–11 years) (Table S12). Across both age groups, the vast majority of OCS and TCS prescriptions were issued by practitioner clinics (Table S13). Although it is possible that TCS were mainly used for other comorbidities such as eczema or atopic dermatitis, the rates of TCS use (children 74.0% and adolescents/adults 51.8%) in this study were notably higher than those of comorbid atopic dermatitis (children 38.7% and adolescents/adults 18.7%). This suggests that TCS were also widely used for CSU in Japan, which is consistent with the results of a web‐based study in Japan showing that 35.0% of adult participants responded that they used TCS as their current medication for CSU [7]. The situation may be similar in the US, as a study using the US HealthVerity claims database recently reported that 49.1% (*n* = 81 621/166 195) of CSU patients were prescribed TCS as treatment for CSU during the follow‐up period (post‐CSU diagnosis) [18].

Regarding trends in treatment steps during the 12‐month follow‐up period, most patients received the same treatment step level as their initial treatment, with Step 2 being the most stable treatment step. More detailed observation of treatment step changes made during the follow‐up period showed that Step 1b patients changed their treatment relatively late (after 4 months) while Step 1a and Step 3 patients included higher percentages of those changing treatment at 2 to 3 months than Step 1b. Step 3 was the second most common treatment step used at the index month, and the proportion of patients at this step dropped most notably at 2 to 3 months, consistent with the change in treatment at this time point. Following this, the proportion of patients at Step 3 remained relatively stable during the follow‐up period. As noted previously, OCS was used relatively often in the chronic phase over omalizumab, suggesting that biological preparations have not been well incorporated into prescribing practices, possibly due to greater ease of OCS prescribing. Findings from the Sankey diagram suggest that some patients continued treatment intermittently, given that approximately one‐third of the patients categorized as having ‘no prescription’ later transitioned back into a treatment group. It should be noted that ‘no prescription’ does not necessarily mean ‘cured’ but includes any reasons for this outcome, such as treatment discontinuation or switching to over‐the‐counter medications.

Regarding HCRU, CSU‐related and all‐cause hospitalizations were notably different, whereas values for outpatient visits were the same in both children and adolescents/adults (Table 2). Patients in Japan tend to receive outpatient treatment for CSU and other conditions at the same facility, whereas hospitalizations for conditions other than CSU would be reflected separately. HCRU associated with CSU shows several differences compared with other common dermatological diseases, such as atopic dermatitis (AD). Based on a retrospective claims database study in Japan concerning patients with AD [29], it appears that the number of outpatient visits with CSU is lower than that of AD. Further, the frequency of hospitalization was slightly higher in the CSU Cohort of this study than that of AD cohorts and matched non‐AD cohorts from the abovementioned analysis. Finally, in the case of AD, most admissions were for short‐term educational purposes, whereas in the case of CSU, admissions for more than a week could occur, although less frequently.

In the present study, 42% of patients remained on omalizumab at 6 months and 16% at 12 months. This observation is somewhat supported by a real‐world study that found that omalizumab continued in approximately 60% of patients aged 18 to 64 years after 24 weeks [30]. A recent multinational observational study on omalizumab drug survival based on the CURE registry demonstrated a 76% drug survival at 1 year on average, and a Japanese subcohort also demonstrated a similar rate [31]. Our present study demonstrates much lower “drug survival” at 1 year, reflecting the real‐world situation in Japan, contrary to the selected sites with high‐level expertise in urticaria management on the CURE registry. The most dominant reason for omalizumab discontinuation in the abovementioned study was reported well‐controlled disease; however, in the present study, we cannot collect the information about the reason for discontinuation from the claims database. A real‐world post‐marketing surveillance study in Japanese patients highlighted the efficacy of omalizumab, finding that 81% and 95% of patients reached UCT ≥ 12 and DLQI ≤ 5, respectively, at Week 52 [11]. However, many patients who initially recovered subsequently relapsed, with 23% of patients requiring retreatment following relapse [11]. In the present study, approximately 25% of patients restarted omalizumab, suggesting that an as‐needed therapy is widely used with omalizumab. Other treatment options to achieve long‐term control without flares in such patients are expected [32].

Key strengths of the present study include the fact that the database used covers about 10% of the Japanese population, including corporate employees and their families from all over Japan. Further, during the period in which a patient is a member of the same health insurance association, they can be traced even if they visit other hospitals or clinics, allowing data on treatment and medical history to be obtained longitudinally.

However, there are several limitations inherent in the database as well as the methodology that affect this study. Firstly, diagnoses provided to the claims database may differ from the actual clinical diagnosis and may not correctly classify the type of urticaria in some patients. We justified the inclusion of L50.8 (other urticaria) in CSU based on the fact that CSU in Japan is defined as the term of “Chronic urticaria” as per the Japanese Treatment Guidelines [3], and “Chronic urticaria” is included in L50.8. Further, although it may include other types other than “Chronic urticaria”, these are considered to be quite rare in Japan, such that they are not included in the main urticaria types in the Japanese Treatment Guidelines [3], and their prevalence is limited in Japan according to a Japanese primary care physician survey [4]. In relation to diagnostic codes, we also acknowledge that the CIndU criteria did not include other CIndU types, such as solar urticaria (L56.3), as well as both non‐allergic urticaria and delayed pressure urticaria (no specific ICD‐10 code). Further, we recognize the inherent limitations in accurately diagnosing CIndU and selecting an insurance disease name of CIndU in clinical practice and acknowledge that some patients might be classified under the general disease name “urticaria” associated with L50.9 (unspecified urticaria). However, we minimized the potential inclusion of CIndU in the CSU Cohort by implementing specific inclusion criteria (antihistamine prescription required for more than 6 weeks in the 3 months after the index month) and, as above, excluded patients with ICD‐10 codes L50.0 (allergic urticaria), L50.2 (urticaria due to cold and heat), L50.3 (dermatografic urticaria), L50.4 (vibratory urticaria), L50.5 (cholinergic urticaria), and L50.6 (contact urticaria), which encompass many types of inducible urticaria. Some physicians may also have categorized non‐allergic urticaria and delayed pressure urticaria as L50.8 (other urticaria) and these issues represent potential limitations of this study. Secondly, some drugs and HCRU items listed in the database may have been provided for purposes other than CSU because the diseases are not linked to drug prescription and medical procedure data, and prescribed drugs may not have been taken by patients as recorded in the claims data. This study also did not include patients who have CSU without any visit to clinics or hospitals for various reasons, such as mild severity, treatment abandonment, or using over‐the‐counter medications. Thirdly, the database can not specify the reason for non‐prescription, such as remission or nonadherence. Further, data on over‐the‐counter drugs could not be collected. More broadly, the effectiveness of both over‐the‐counter and prescribed treatment cannot be assessed through database claims methodology. Finally, the number of subjects aged over 65 years was limited due to retirement of employment, and subjects aged over 75 years were not included because such patients receive national medical insurance.

## 5 Conclusion

This study using the JMDC claims database found that the prevalence of CSU in the Japanese population has been gradually increasing despite no notable increase in incidence in new‐onset cases. Comorbid allergic disorders are common in this population, and OCS were broadly used, even in children. Many patients continue to receive the same guideline‐based intensity step treatment for a long period. Medical resource utilization by CSU patients in Japan was also described. These features suggest that further treatment options for uncontrolled patients are needed to improve symptoms, reduce the possibility of unwanted adverse effects as well as reduce the burden on medical resources.

## Disclosure

*Approval of the Research Protocol by an Institutional Reviewer Board*: Although this study used anonymized data and did not collect, use, or transmit individually identifiable patient data, the study sponsor submitted the study protocol to the Institutional Review Board for review and approval.

*Animal Studies*: N/A.

## Consent

The authors have nothing to report.

## Conflicts of Interest

A.F. has received research funding from Taiho Pharmaceutical Co. Ltd. and lecture fees from Novartis Pharma K.K., Sanofi K.K., Kyowa Kirin Co. Ltd., KYORIN Pharmaceutical Co. Ltd., Mitsubishi Tanabe Pharma Corporation, and Taiho Pharmaceutical Co. Ltd. Y.K., Y.S., and K.A. are employees of Sanofi K.K. and may hold shares in the company.

## Supporting information


**Data S1:** jde17943‐sup‐0001‐DataS1.docx.

## Data Availability

The data that support the findings of this study are available from the corresponding author upon reasonable request.
