# Supplementary material for: Prevalence, Comorbidities, and Current Management of Chronic Spontaneous Urticaria in Japan: Retrospective Claims Database Study
Source: J Dermatol. 2025 Sep 8;52(12):1775–87. doi: 10.1111/1346-8138.17943 (PMC12698888; doi:10.1111/1346-8138.17943)

# Supporting Information

**Table S1.** Comorbidities observed at least once during baseline period (ICD-10 diagnosis code)

| Comorbidities were observed at least one diagnosis during baseline period using the appropriate ICD-10 diagnosis codes as follows:   - Allergic diseases (Any of the following “Atopic dermatitis” to “Eosinophilic esophagitis” diagnosis)   - Atopic dermatitis   - Asthma   - Allergic rhinitis   - Food allergy   - Chronic rhinosinusitis with nasal polyp (J32 or J33)   - Chronic rhinosinusitis (J32)   - Nasal polyp (J33)   - Chronic rhinosinusitis with nasal polyp (J32 and J33 at the same month)   - Conjunctivitis   - Prurigo   - Allergic contact dermatitis, drug allergy, metal allergy (L23)   - Allergic contact dermatitis due to metals (L230)   - Allergic contact dermatitis due to adhesives (L231)   - Allergic contact dermatitis due to cosmetics (L232)   - Allergic contact dermatitis due to drugs in contact with skin (L233)   - Allergic contact dermatitis due to dyes (L234)   - Allergic contact dermatitis due to other chemical products (L235)   - Allergic contact dermatitis due to plants, except food (L237)   - Allergic contact dermatitis due to other agents (L238)   - Allergic contact dermatitis, unspecified cause (L239)   - Eosinophilic esophagitis - Hereditary angioedema, acquired angioedema - Hypothyroidism - Thyroiditis - Rheumatoid arthritis - Systemic lupus erythematosus - Sjögren syndrome - Celiac disease - Type 1 diabetes - Vitiligo - Migraine - Chronic Inducible Urticaria   In addition, all diseases diagnosed at least one time during baseline period are extracted in ICD-10 level 4 classification and disease name. |
| --- |

**Table S2.** Prescription type counted during follow-up period according to the Japanese Treatment Guidelines (2018)

| **Step 1**   - H_1_-antihistamines   - Minimally- or non-sedating second-generation H_1_-antihistamines   Step 1a; standard dose, Step 1b; up-dosing or combination  **Step 2**   - H_2_-antihistamines - Antileukotrienes - Vaccinia virus inoculated house rabbit inflammatory skin extract (injection) - Glycyrrhizin preparation (injection) - Diphenyl sulfone - Anti-anxiety drug - Tranexamic acid - Chinese herbal medicine   **Step 3**   - Steroid   - Oral corticosteroids (OCS) and Topical corticosteroids (TCS) - Omalizumab - Immunosuppressant   - Cyclosporine |
| --- |

**Table S3.** Breakdown of health insurance disease names specified in Japan, associated with each ICD-10 code, for CSU Cohort

| **ICD-10 code, n (%)***  　Health insurance disease name, n (%)* | **CSU Cohort (N=76710)** | |
| --- | --- | --- |
|  | **0–11 years (N=19665)** | **12–74 years (N=57045)** |
| L50.9 (Urticaria, unspecified) | 18955 (96.4) | 49905 (87.5) |
| Urticaria | 18955 (96.4) | 49905 (87.5) |
| L50.8 (Other urticaria) | 808 (4.1) | 8116 (14.2) |
| Chronic urticaria | 804 (4.1) | 8072 (14.2) |
| Drug-induced urticaria | 2 (<0.1) | 39 (<0.1) |
| Autoimmune urticaria | 1 (<0.1) | 3 (<0.1) |
| Cyclic recurrent urticaria | 1 (<0.1) | 3 (<0.1) |
| Hemorrhagic urticaria | 0 (0) | 2 (<0.1) |
| Familial cold autoinflammatory syndrome | 0 (0) | 0 (0) |
| Aspirin urticaria | 0 (0) | 0 (0) |
| L50.1 (Idiopathic urticaria) | 103 (0.5) | 196 (0.3) |
| Idiopathic urticaria | 103 (0.5) | 196 (0.3) |

*Patients with multiple codes and/or disease names were counted multiple times.

**Table S4.** Prevalence in CSU population

|  |  | **Year** | **2016** | **2017** | **2018** | **2019** | **2020** | **2021** |
| --- | --- | --- | --- | --- | --- | --- | --- | --- |
| Overall |  | Number of patients (n) | 46106 | 60115 | 85059 | 100032 | 112478 | 121666 |
|  |  | Population size (n) | 3707178 | 4682608 | 6047937 | 6590942 | 7377045 | 7780239 |
|  |  | Prevalence (95% CI) | 1.2 (1.2, 1.3) | 1.3 (1.3, 1.3) | 1.4 (1.4, 1.4) | 1.5 (1.5, 1.5) | 1.5 (1.5, 1.5) | 1.6 (1.6, 1.6) |
| Age | 0-9 | Number of patients | 11247 | 13874 | 18927 | 22001 | 20484 | 21825 |
|  |  | Population size | 538123 | 655455 | 822240 | 884936 | 961451 | 984444 |
|  |  | Prevalence (95% CI) | 2.1 (2.1, 2.1) | 2.1 (2.1, 2.2) | 2.3 (2.3, 2.3) | 2.5 (2.5, 2.5) | 2.1 (2.1, 2.2) | 2.2 (2.2, 2.2) |
|  | 10-19 | Number of patients | 5108 | 6975 | 9831 | 11814 | 13824 | 15390 |
|  |  | Population size | 513401 | 655228 | 837739 | 907247 | 998025 | 1055920 |
|  |  | Prevalence (95% CI) | 1.0 (1.0, 1.0) | 1.1 (1.0, 1.1) | 1.2 (1.2, 1.2) | 1.3 (1.3, 1.3) | 1.4 (1.4, 1.4) | 1.5 (1.4, 1.5) |
|  | 20-29 | Number of patients | 3733 | 4600 | 6749 | 8091 | 10340 | 11207 |
|  |  | Population size | 475998 | 584672 | 767049 | 848460 | 982807 | 1027270 |
|  |  | Prevalence (95% CI) | 0.8 (0.8, 0.8) | 0.8 (0.8, 0.8) | 0.9 (0.9, 0.9) | 1.0 (0.9, 1.0) | 1.1 (1.0, 1.1) | 1.1 (1.1, 1.1) |
|  | 30-39 | Number of patients | 6640 | 8209 | 11737 | 13505 | 15579 | 15983 |
|  |  | Population size | 657746 | 790037 | 1014615 | 1086407 | 1199972 | 1222439 |
|  |  | Prevalence (95% CI) | 1.0 (1.0, 1.0) | 1.0 (1.0, 1.1) | 1.2 (1.1, 1.2) | 1.2 (1.2, 1.3) | 1.3 (1.3, 1.3) | 1.3 (1.3, 1.3) |
|  | 40-49 | Number of patients | 9523 | 12892 | 18205 | 21022 | 24023 | 25025 |
|  |  | Population size | 762695 | 990525 | 1266603 | 1359079 | 1495009 | 1542494 |
|  |  | Prevalence (95% CI) | 1.2 (1.2, 1.3) | 1.3 (1.3, 1.3) | 1.4 (1.4, 1.5) | 1.5 (1.5, 1.6) | 1.6 (1.6, 1.6) | 1.6 (1.6, 1.6) |
|  | 50-59 | Number of patients | 7084 | 9574 | 14199 | 17235 | 20372 | 23004 |
|  |  | Population size | 529137 | 692724 | 949489 | 1068418 | 1227084 | 1362618 |
|  |  | Prevalence (95% CI) | 1.3 (1.3, 1.4) | 1.4 (1.4, 1.4) | 1.5 (1.5, 1.5) | 1.6 (1.6, 1.6) | 1.7 (1.6, 1.7) | 1.7 (1.7, 1.7) |
|  | 60-69 | Number of patients | 2447 | 3560 | 4775 | 5595 | 6869 | 8022 |
|  |  | Population size | 206293 | 280462 | 347823 | 387431 | 449843 | 509637 |
|  |  | Prevalence (95% CI) | 1.2 (1.1, 1.2) | 1.3 (1.2, 1.3) | 1.4 (1.3, 1.4) | 1.4 (1.4, 1.5) | 1.5 (1.5, 1.6) | 1.6 (1.5, 1.6) |
|  | ≥70 | Number of patients | 324 | 431 | 636 | 769 | 987 | 1210 |
|  |  | Population size | 23785 | 33505 | 42379 | 48964 | 62854 | 75417 |
|  |  | Prevalence (95% CI) | 1.4 (1.2, 1.5) | 1.3 (1.2, 1.4) | 1.5 (1.4, 1.6) | 1.6 (1.5, 1.7) | 1.6 (1.5, 1.7) | 1.6 (1.5, 1.7) |
| Gender | Male | Number of patients | 21793 | 28476 | 38917 | 45112 | 50116 | 54037 |
|  |  | Population size | 2069233 | 2614823 | 3322998 | 3605457 | 4002201 | 4264832 |
|  |  | Prevalence (95% CI) | 1.1 (1.0, 1.1) | 1.1 (1.1, 1.1) | 1.2 (1.2, 1.2) | 1.3 (1.2, 1.3) | 1.3 (1.2, 1.3) | 1.3 (1.3, 1.3) |
|  | Female | Number of patients | 24313 | 31639 | 46142 | 54920 | 62362 | 67629 |
|  |  | Population size | 1637945 | 2067785 | 2724939 | 2985485 | 3374844 | 3515407 |
|  |  | Prevalence (95% CI) | 1.5 (1.5, 1.5) | 1.5 (1.5, 1.5) | 1.7 (1.7, 1.7) | 1.8 (1.8, 1.9) | 1.8 (1.8, 1.9) | 1.9 (1.9, 1.9) |

Abbreviations: CI, confidence interval

**Table S5.** Incidence in CSU population

|  |  | **Year** | **2016** | **2017** | **2018** | **2019** | **2020** | **2021** |
| --- | --- | --- | --- | --- | --- | --- | --- | --- |
| Overall |  | Newly diagnosed patients (n) | 16905 | 25126 | 32801 | 46343 | 49520 | 51996 |
|  |  | Population size (n) | 2387382 | 3400882 | 4219723 | 5502250 | 6008592 | 6541745 |
|  |  | Incidence (%) (95% CI) | 0.7 (0.7, 0.7) | 0.7 (0.7, 0.7) | 0.8 (0.8, 0.8) | 0.8 (0.8, 0.8) | 0.8 (0.8, 0.8) | 0.8 (0.8, 0.8) |
| Age | 0-9 | Newly diagnosed patients | 5070 | 7269 | 9321 | 12740 | 11225 | 12123 |
|  |  | Population size | 326465 | 457689 | 547772 | 690952 | 742608 | 786007 |
|  |  | Incidence (%) (95% CI) | 1.6 (1.5, 1.6) | 1.6 (1.6, 1.6) | 1.7 (1.7, 1.7) | 1.8 (1.8, 1.9) | 1.5 (1.5, 1.5) | 1.5 (1.5, 1.6) |
|  | 10-19 | Newly diagnosed patients | 2314 | 3389 | 4654 | 6566 | 7459 | 7849 |
|  |  | Population size | 353211 | 485056 | 607715 | 780349 | 843177 | 900534 |
|  |  | Incidence (%) (95% CI) | 0.7 (0.6, 0.7) | 0.7 (0.7, 0.7) | 0.8 (0.7, 0.8) | 0.8 (0.8, 0.9) | 0.9 (0.9, 0.9) | 0.9 (0.9, 0.9) |
|  | 20-29 | Newly diagnosed patients | 1199 | 1783 | 2250 | 3357 | 4354 | 4786 |
|  |  | Population size | 273371 | 392984 | 475357 | 627670 | 706614 | 787958 |
|  |  | Incidence (%) (95% CI) | 0.4 (0.4, 0.5) | 0.5 (0.4, 0.5) | 0.5 (0.5, 0.5) | 0.5 (0.5, 0.6) | 0.6 (0.6, 0.6) | 0.6 (0.6, 0.6) |
|  | 30-39 | Newly diagnosed patients | 2243 | 3450 | 4217 | 6050 | 6874 | 6931 |
|  |  | Population size | 408548 | 595755 | 703001 | 910804 | 977528 | 1055274 |
|  |  | Incidence (%) (95% CI) | 0.5 (0.5, 0.6) | 0.6 (0.6, 0.6) | 0.6 (0.6, 0.6) | 0.7 (0.6, 0.7) | 0.7 (0.7, 0.7) | 0.7 (0.6, 0.7) |
|  | 40-49 | Newly diagnosed patients | 3092 | 4668 | 6122 | 8595 | 9437 | 9473 |
|  |  | Population size | 511048 | 737050 | 907422 | 1175121 | 1263272 | 1344307 |
|  |  | Incidence (%) (95% CI) | 0.6 (0.6, 0.6) | 0.6 (0.6, 0.7) | 0.7 (0.7, 0.7) | 0.7 (0.7, 0.7) | 0.7 (0.7, 0.8) | 0.7 (0.7, 0.7) |
|  | 50-59 | Newly diagnosed patients | 2159 | 3234 | 4417 | 6632 | 7396 | 7758 |
|  |  | Population size | 370766 | 513240 | 686630 | 938941 | 1052229 | 1181213 |
|  |  | Incidence (%) (95% CI) | 0.6 (0.6, 0.6) | 0.6 (0.6, 0.7) | 0.6 (0.6, 0.7) | 0.7 (0.7, 0.7) | 0.7 (0.7, 0.7) | 0.7 (0.6, 0.7) |
|  | 60-69 | Newly diagnosed patients | 718 | 1206 | 1598 | 2095 | 2429 | 2661 |
|  |  | Population size | 127659 | 197445 | 258980 | 335193 | 371889 | 424761 |
|  |  | Incidence (%) (95% CI) | 0.6 (0.5, 0.6) | 0.6 (0.6, 0.6) | 0.6 (0.6, 0.6) | 0.6 (0.6, 0.7) | 0.7 (0.6, 0.7) | 0.6 (0.6, 0.7) |
|  | ≥70 | Newly diagnosed patients | 110 | 127 | 222 | 308 | 346 | 415 |
|  |  | Population size | 16314 | 21663 | 32846 | 43220 | 51275 | 61691 |
|  |  | Incidence (%) (95% CI) | 0.7 (0.6, 0.8) | 0.6 (0.5, 0.7) | 0.7 (0.6, 0.8) | 0.7 (0.6, 0.8) | 0.7 (0.6, 0.7) | 0.7 (0.6, 0.7) |
| Gender | Male | Newly diagnosed patients | 8002 | 11854 | 15175 | 21002 | 22298 | 22813 |
|  |  | Population size | 1365812 | 1922951 | 2386424 | 3065776 | 3329409 | 3613642 |
|  |  | Incidence (%) (95% CI) | 0.6 (0.6, 0.6) | 0.6 (0.6, 0.6) | 0.6 (0.6, 0.6) | 0.7 (0.7, 0.7) | 0.7 (0.7, 0.7) | 0.6 (0.6, 0.6) |
|  | Female | Newly diagnosed patients | 8903 | 13272 | 17626 | 25341 | 27222 | 29183 |
|  |  | Population size | 1021570 | 1477931 | 1833299 | 2436474 | 2679183 | 2928103 |
|  |  | Incidence (%) (95% CI) | 0.9 (0.9, 0.9) | 0.9 (0.9, 0.9) | 1.0 (0.9, 1.0) | 1.0 (1.0, 1.1) | 1.0 (1.0, 1.0) | 1.0 (1.0, 1.0) |

Abbreviations: CI, confidence interval

**Table S6.** Characteristics of H_1_-antihistamines treatment

| **H_1_-antihistamines prescription regardless of other treatments** | **CSU Cohort (N=76710)** |
| --- | --- |
| **Prescription days, days/year, median (Q1, Q3)** |  |
| Children (0-11 years) | 170.6 (91.1, 276.2) |
| Adolescents/adults (12-74 years) | 159.7 (71.0, 292.4) |
| **Number of combination prescriptions, yes, n (%)** |  |
| Children (0-11 years) | 2608 (13.3) |
| Adolescents/adults (12-74 years) | 3732 (6.5) |
| **Number of double prescriptions, yes, n (%)** |  |
| Children (0-11 years) | 2776 (14.1) |
| Adolescents/adults (12-74 years) | 9738 (17.1) |
| **Long-term maintenance of Step 1a (standard dose of single H1-antihistamine only)** | |
| n (%) | 25900 (33.8) |
| Prescription duration, days/year |  |
| Mean (SD) | 159.7 (101.8) |
| Median (Q1, Q3) | 119.0 (76.0, 229.0) |

Abbreviations: Q1, Q3 = interquartile range; SD, standard deviation.

**Table S7.** Prescription dose of topical and oral corticosteroids

|  | | **0-11 years (N=19965)** | | **12-74 year (N=57045)** | |
| --- | --- | --- | --- | --- | --- |
| Topical corticosteroids | |  |  |  |  |
|  | Cumulative dose (g/year) |  |  |  |  |
|  | Strongest |  |  |  |  |
|  | N, % | 635 | (3.2) | 5126 | (9.0) |
|  | 1Q | 5.0 |  | 10.0 |  |
|  | Median | 10.0 |  | 30.0 |  |
|  | 3Q | 23.0 |  | 80.1 |  |
|  | Very strong |  |  |  |  |
|  | N, % | 4279 | (21.8) | 17964 | (31.5) |
|  | 1Q | 10.0 |  | 15.0 |  |
|  | Median | 20.0 |  | 35.0 |  |
|  | 3Q | 49.0 |  | 100.1 |  |
|  | Strong |  |  |  |  |
|  | N, % | 9994 | (50.8) | 14653 | (25.7) |
|  | 1Q | 10.0 |  | 10.0 |  |
|  | Median | 20.0 |  | 20.0 |  |
|  | 3Q | 50.0 |  | 54.9 |  |
|  | Medium |  |  |  |  |
|  | N, % | 9971 | (50.7) | 11006 | (19.3) |
|  | 1Q | 10.0 |  | 5.0 |  |
|  | Median | 25.0 |  | 13.7 |  |
|  | 3Q | 66.0 |  | 34.9 |  |
|  | Weak |  |  |  |  |
|  | N, % | 72 | (0.4) | 104 | (0.2) |
|  | 1Q | 5.0 |  | 5.0 |  |
|  | Median | 10.0 |  | 10.0 |  |
|  | 3Q | 20.6 |  | 46.5 |  |
| Cumulative dose of oral corticosteroids (mg/year) | |  |  |  |  |
|  | N, % | 3270 | (16.6) | 11580 | (20.3) |
|  | 1Q | 13.3 |  | 35.0 |  |
|  | Median | 26.7 |  | 80.1 |  |
|  | 3Q | 57.0 |  | 225.2 |  |
| Prescription duration of oral corticosteroids (days/year) | |  |  |  |  |
|  | N, % | 3270 | (16.6) | 11580 | (20.3) |
|  | 1Q | 2.0 |  | 5.0 |  |
|  | Median | 5.0 |  | 14.0 |  |
|  | 3Q | 14.0 |  | 58.0 |  |
| Dose of OCS (mg) | |  |  |  |  |
|  | N, % | 3270 | (16.6) | 11580 | (20.3) |
|  | 1Q | 2.5 |  | 2.5 |  |
|  | Median | 5.0 |  | 5.0 |  |
|  | 3Q | 10.0 |  | 10.0 |  |

Abbreviations: Q1, Q3 = interquartile range.

**Table S8.** Characteristics of omalizumab treatment in terms of survival and restart

| **Characteristic** | **Omalizumab Cohort (N = 81)** |
| --- | --- |
| Persistence, n (%) |  |
| At 6 months | 34 (42.0) |
| At 12 months | 13 (16.0) |
| Restarting after discontinuation |  |
| Time to restart, days |  |
| Mean (SD) | 123.7 (59.5) |
| Median (Q1, Q3) | 112.0 (79.0, 141.0) |

Abbreviations: Q1, Q3 = interquartile range; SD, standard deviation.

**Table S9.** Prevalence in CIndU populations

Patients with CIndU are defined as those (i) diagnosed with following urticaria ICD-10 codes: L50.0 [Allergic urticaria], L50.2 [Urticaria due to cold and heat], L50.3 [Dermatographic urticaria], L50.4 [Vibrating urticaria], L50.5 [Cholinergic urticaria], L50.6 [Contact urticaria], and (ii) prescribed H_1_-antihistamines for more than 6 weeks within a 3-month period.

|  |  | **Cohort (Year)** | **CIndU (2021)** |
| --- | --- | --- | --- |
| Overall |  | Number of patients (n) | 4734 |
|  |  | Population size (n) | 7780239 |
|  |  | Prevalence (95% CI) | <0.1 (<0.1, <0.1) |
| Age | 0-9 | Number of patients | 899 |
|  |  | Population size | 984444 |
|  |  | Prevalence (95% CI) | <0.1 (<0.1, <0.1) |
|  | 10-19 | Number of patients | 720 |
|  |  | Population size | 1055920 |
|  |  | Prevalence (95% CI) | <0.1 (<0.1, <0.1) |
|  | 20-29 | Number of patients | 500 |
|  |  | Population size | 1027270 |
|  |  | Prevalence (95% CI) | <0.1 (<0.1, <0.1) |
|  | 30-39 | Number of patients | 585 |
|  |  | Population size | 1222439 |
|  |  | Prevalence (95% CI) | <0.1 (<0.1, <0.1) |
|  | 40-49 | Number of patients | 787 |
|  |  | Population size | 1542494 |
|  |  | Prevalence (95% CI) | <0.1 (<0.1, <0.1) |
|  | 50-59 | Number of patients | 836 |
|  |  | Population size | 1362618 |
|  |  | Prevalence (95% CI) | <0.1 (<0.1, <0.1) |
|  | 60-69 | Number of patients | 348 |
|  |  | Population size | 509637 |
|  |  | Prevalence (95% CI) | <0.1 (<0.1, <0.1) |
|  | ≥70 | Number of patients | 59 |
|  |  | Population size | 75417 |
|  |  | Prevalence (95% CI) | <0.1 (<0.1, 0.1) |
| Gender | Male | Number of patients | 2191 |
|  |  | Population size | 4264832 |
|  |  | Prevalence (95% CI) | <0.1 (<0.1, <0.1) |
|  | Female | Number of patients | 2543 |
|  |  | Population size | 3515407 |
|  |  | Prevalence (95% CI) | <0.1 (<0.1, <0.1) |

Abbreviations: CI, confidence interval; CIndU, chronic inducible urticaria.

**Table S10.** Breakdown of medical departments in CSU Cohort at index month

|  | **CSU Cohort**  **(without imputation*)** | |  | **CSU Cohort**  **(with imputation*)** | |
| --- | --- | --- | --- | --- | --- |
| **Department, n (%)**** | **0-11 years  (N = 3865)** | **12-74 years (N=10513)** |  | **0-11 years  (N = 19665)** | **12-74 years (N=57045)** |
| Dermatology | 887 (22.9) | 5788 (55.1) |  | 6992 (35.6) | 34444 (60.4) |
| General internal medicine | 513 (13.3) | 2736 (26.0) |  | 3664 (18.6) | 15150 (26.6) |
| Pediatrics | 2327 (60.2) | 449 (4.3) |  | 8515 (43.3) | 1985 (3.5) |
| Urology | 1 (0.0) | 20 (0.2) |  | 129 (0.7) | 1082 (1.9) |
| Otolaryngology | 41 (1.1) | 196 (1.9) |  | 477 (2.4) | 968 (1.7) |
| General surgery | 12 (0.3) | 301 (2.9) |  | 105 (0.5) | 894 (1.6) |
| Plastic surgery | 1 (0.0) | 38 (0.4) |  | 150 (0.8) | 860 (1.5) |
| Orthopedics | 15 (0.4) | 161 (1.5) |  | 82 (0.4) | 705 (1.2) |
| Gastroenterology | 4 (0.1) | 164 (1.6) |  | 46 (0.2) | 469 (0.8) |
| Obstetrics and gynecology | 3 (0.1) | 167 (1.6) |  | 75 (0.4) | 405 (0.7) |
| Other Departments | 0 (0.0) | 0 (0.0) |  | 0 (0.0) | 248 (0.4) |
| Cardiology | 2 (0.1) | 81 (0.8) |  | 16 (0.1) | 195 (0.3) |
| Department of Allergology | 59 (1.5) | 49 (0.5) |  | 122 (0.6) | 192 (0.3) |
| Psychiatry | 3 (0.1) | 65 (0.6) |  | 9 (0.0) | 189 (0.3) |
| Ophthalmology | 9 (0.2) | 53 (0.5) |  | 40 (0.2) | 178 (0.3) |
| Pulmonology | 0 (0.0) | 79 (0.8) |  | 6 (0.0) | 176 (0.3) |
| Pychosomatic medicine | 0 (0.0) | 8 (0.1) |  | 5 (0.0) | 112 (0.2) |
| Neurosurgery | 3 (0.1) | 49 (0.5) |  | 10 (0.1) | 106 (0.2) |
| Emergency department | 31 (0.8) | 84 (0.8) |  | 33 (0.2) | 86 (0.2) |
| Neurology | 1 (0.0) | 43 (0.4) |  | 2 (0.0) | 64 (0.1) |
| Other Internal Medicine | 0 (0.0) | 0 (0.0) |  | 6 (0.0) | 42 (0.1) |
| Rheumatology | 0 (0.0) | 33 (0.3) |  | 0 (0.0) | 41 (0.1) |
| Anesthesiology | 0 (0.0) | 4 (0.0) |  | 13 (0.1) | 31 (0.1) |
| Department of Metabolism, Endocrinology and Diabetes | 0 (0.0) | 0 (0.0) |  | 0 (0.0) | 21 (0.0) |
| Other Surgeries | 0 (0.0) | 0 (0.0) |  | 0 (0.0) | 21 (0.0) |
| Radiology | 0 (0.0) | 12 (0.1) |  | 4 (0.0) | 21 (0.0) |
| Cardiovascular surgery | 1 (0.0) | 13 (0.1) |  | 1 (0.0) | 15 (0.0) |
| Neuropsychiatry | 1 (0.0) | 4 (0.0) |  | 1 (0.0) | 11 (0.0) |
| Dentistry | 0 (0.0) | 0 (0.0) |  | 11 (0.1) | 11 (0.0) |
| Department of Rehabilitation | 3 (0.1) | 6 (0.1) |  | 16 (0.1) | 9 (0.0) |
| Nephrology | 0 (0.0) | 0 (0.0) |  | 2 (0.0) | 8 (0.0) |
| Respiratory surgery | 0 (0.0) | 4 (0.0) |  | 0 (0.0) | 7 (0.0) |

*Missing information on medical departments was imputed using the primary medical department listed for each institution.

**Patients visiting multiple medical institutes at index month were counted multiple times.

**Table S11.** Breakdown of departments (practitioner and hospital doctor) in CSU Cohort at index month

|  | **CSU Cohort**  **(without imputation*)** | |  | **CSU Cohort**  **(with imputation*)** | |
| --- | --- | --- | --- | --- | --- |
| **Department, n (%)**** | **0-11 years  (N = 3865)** | **12-74 years  (N = 10513)** |  | **0-11 years  (N = 19665)** | **12-74 years  (N = 57045)** |
| All departments |  |  |  |  |  |
| Practitioner | 1787 (46.2) | 5132 (48.8) |  | 17666 (89.8) | 51511 (90.3) |
| Hospital doctor | 2142 (55.4) | 5486 (52.2) |  | 2350 (12.0) | 6312 (11.1) |
| Dermatology | 887 | 5788 |  | 6992 | 34444 |
| Practitioner | 623 (70.2) | 3063 (52.9) |  | 6749 (96.5) | 31911 (92.6) |
| Hospital doctor | 268 (30.2) | 2749 (47.5) |  | 268 (3.8) | 2749 (8.0) |
| General internal medicine | 513 | 2736 |  | 3664 | 15150 |
| Practitioner | 487 (94.9) | 1571 (57.4) |  | 3481 (95.0) | 13415 (88.5) |
| Hospital doctor | 27 (5.3) | 1175 (42.9) |  | 191 (5.2) | 1817 (12.0) |
| Otolaryngology | 41 | 196 |  | 477 | 968 |
| Practitioner | 23 (56.1) | 90 (45.9) |  | 459 (96.2) | 862 (89.0) |
| Hospital doctor | 18 (43.9) | 106 (54.1) |  | 18 (3.8) | 106 (11.0) |
| Pediatrics | 2327 | 449 |  | 8515 | 1985 |
| Practitioner | 635 (27.3) | 150 (33.4) |  | 6909 (81.1) | 1693 (85.3) |
| Hospital doctor | 1715 (73.7) | 300 (66.8) |  | 1719 (20.2) | 302 (15.2) |
| Orthopedics | 15 | 161 |  | 82 | 705 |
| Practitioner | 6 (40.0) | 60 (37.3) |  | 60 (73.2) | 518 (73.5) |
| Hospital doctor | 9 (60.0) | 101 (62.7) |  | 22 (26.8) | 187 (26.5) |
| Urology | 129 | 1082 |  | 129 | 1082 |
| Practitioner | 1 (100.0) | 20 (25.0) |  | 129 (100.0) | 1020 (94.3) |
| Hospital doctor | 0 (0.0) | 60 (75.0) |  | 0 (0.0) | 63 (5.8) |
| General surgery | 12 | 301 |  | 105 | 894 |
| Practitioner | 2 (16.7) | 55 (18.3) |  | 90 (85.7) | 605 (67.7) |
| Hospital doctor | 10 (83.3) | 247 (82.1) |  | 15 (14.3) | 291 (32.6) |
| Plastic surgery | 1 | 38 |  | 150 | 860 |
| Practitioner | 1 (100.0) | 14 (36.8) |  | 150 (100.0) | 834 (97.0) |
| Hospital doctor | 0 (0.0) | 24 (63.2) |  | 0 (0.0) | 26 (3.0) |
| Obstetrics and gynecology | 3 | 167 |  | 75 | 405 |
| Practitioner | 2 (66.7) | 24 (14.4) |  | 65 (86.7) | 247 (61.0) |
| Hospital doctor | 1 (33.3) | 143 (85.6) |  | 10 (13.3) | 159 (39.3) |
| Pulmonology | 0 | 79 |  | 6 | 176 |
| Practitioner | – | 4 (5.1) |  | 6 (100.0) | 94 (53.4) |
| Hospital doctor | – | 75 (94.9) |  | 0 (0.0) | 82 (46.6) |
| Gastroenterology | 4 | 164 |  | 46 | 469 |
| Practitioner | 3 (75.0) | 35 (21.3) |  | 43 (93.5) | 337 (71.9) |
| Hospital doctor | 1 (25.0) | 130 (79.3) |  | 3 (6.5) | 133 (28.4) |
| Ophthalmology | 9 | 53 |  | 40 | 178 |
| Practitioner | 5 (55.6) | 7 (13.2) |  | 36 (90.0) | 132 (74.2) |
| Hospital doctor | 4 (44.4) | 46 (86.8) |  | 4 (10.0) | 46 (25.8) |
| Department of Allergology | 59 | 49 |  | 122 | 192 |
| Practitioner | 2 (3.4) | 1 (2.0) |  | 65 (53.3) | 144 (75.0) |
| Hospital doctor | 57 (96.6) | 48 (98.0) |  | 57 (46.7) | 48 (25.0) |
| Neurosurgery | 3 | 49 |  | 10 | 106 |
| Practitioner | 0 (0.0) | 5 (10.2) |  | 7 (70.0) | 53 (50.0) |
| Hospital doctor | 3 (100.0) | 44 (89.8) |  | 3 (30.0) | 53 (50.0) |
| Cardiology | 2 | 81 |  | 16 | 195 |
| Practitioner | 2 (100.0) | 14 (17.3) |  | 16 (100.0) | 127 (65.1) |
| Hospital doctor | 0 (0.0) | 67 (82.7) |  | 0 (0.0) | 68 (34.9) |
| Rheumatology | 0 | 33 |  | 0 | 41 |
| Practitioner | – | 6 (18.2) |  | 0 (0.0) | 14 (34.1) |
| Hospital doctor | – | 27 (81.8) |  | 0 (0.0) | 27 (65.9) |
| Psychiatry | 3 | 65 |  | 9 | 189 |
| Practitioner | 0 (0.0) | 16 (24.6) |  | 5 (55.6) | 126 (66.7) |
| Hospital doctor | 3 (100.0) | 49 (75.4) |  | 4 (44.4) | 63 (33.3) |
| Neurology | 1 | 43 |  | 2 | 64 |
| Practitioner | 1 (100.0) | 2 (4.7) |  | 2 (100.0) | 20 (31.3) |
| Hospital doctor | 0 | 41 (95.3) |  | 0 (0.0) | 44 (68.8) |
| Psychosomatic medicine | 0 | 8 |  | 5 | 112 |
| Practitioner | – | 5 (26.5) |  | 5 (100.0) | 109 (97.3) |
| Hospital doctor | – | 3 (37.5) |  | 0 (0.0) | 3 (2.7) |
| Other Surgeries | 0 | 0 |  | 0 | 21 |
| Practitioner | – | – |  | 0 (0.0) | 20 (95.2) |
| Hospital doctor | – | – |  | 0 (0.0) | 1 (4.8) |
| Other Departments | 0 | 0 |  | 0 | 248 |
| Practitioner | – | – |  | 0 (0.0) | 248 (100.0) |
| Hospital doctor | – | – |  | 0 (0.0) | 0 (0.0) |
| Department of Metabolism, Endocrinology and Diabetes | 0 | 0 |  | 0 | 21 |
| Practitioner | – | – |  | – | 20 (95.2) |
| Hospital doctor | – | – |  | – | 1 (4.8) |
| Other Internal Medicine | 0 | 0 |  | 6 | 42 |
| Practitioner | – | – |  | 6 (100.0) | 41 (97.6) |
| Hospital doctor | – | – |  | 0 (0.0) | 1 (2.4) |
| Respiratory surgery | 0 | 4 |  | 0 | 7 |
| Practitioner | – | 0 (0.0) |  | – | 1 (14.3) |
| Hospital doctor | – | 4 (100.0) |  | – | 6 (85.7) |
| Anesthesiology | 0 | 4 |  | 13 | 31 |
| Practitioner | – | 0 (0.0) |  | 13 (100.0) | 27 (87.1) |
| Hospital doctor | – | 4 (100.0) |  | 0 (0.0) | 4 (12.9) |
| Radiology | 0 | 12 |  | 4 | 21 |
| Practitioner | – | 1 (8.3) |  | 4 (100.0) | 9 (42.9) |
| Hospital doctor | – | 11 (91.7) |  | 0 (0.0) | 12 (57.1) |
| Emergency department | 31 | 84 |  | 33 | 86 |
| Practitioner | 0 (0.0) | 1 (1.2) |  | 2 (6.1) | 1 (1.2) |
| Hospital doctor | 31 (100.0) | 83 (98.8) |  | 31 (93.9) | 85 (98.8) |
| Dentistry | 0 | 0 |  | 11 | 11 |
| Practitioner | – | – |  | 10 (90.9) | 11 (100.0) |
| Hospital doctor | – | – |  | 1 (9.1) | 0 (0.0) |
| Cardiovascular surgery | 1 | 13 |  | 1 | 15 |
| Practitioner | 0 (0.0) | 1 (7.7) |  | 0 (0.0) | 1 (6.7) |
| Hospital doctor | 1 (100.0) | 12 (92.3) |  | 1 (100.0) | 14 (93.3) |
| Nephrology | 0 | 0 |  | 2 | 8 |
| Practitioner | – | – |  | 2 (100.0) | 8 (100.0) |
| Hospital doctor | – | – |  | 0 (0.0) | 0 (0.0) |
| Neuropsychiatry | 3 | 65 |  | 1 | 11 |
| Practitioner | 0 | 16 (24.6) |  | 0 (0.0) | 10 (90.9) |
| Hospital doctor | 3 (100.0) | 49 (75.4) |  | 1 (100.0) | 1 (9.1) |
| Department of Rehabilitation | 3 | 6 |  | 16 | 9 |
| Practitioner | 0 (0.0) | 1 (16.7) |  | 0 (0.0) | 4 (4.4) |
| Hospital doctor | 3 (100.0) | 5 (83.3) |  | 16 (100.0) | 5 (55.6) |

*Missing information on medical departments was imputed using the primary medical department listed for each institution.

**Patients visiting multiple medical institutes at index month were counted multiple times.

**Table S12.** Breakdown of departments prescribing OCS/TCS in CSU Cohort during the follow-up period

|  | **OCS**  **(without imputation*)** | |  | **TCS**  **(without imputation*)** | |  | **OCS**  **(with imputation*)** | |  | **TCS**  **(with imputation*)** | |
| --- | --- | --- | --- | --- | --- | --- | --- | --- | --- | --- | --- |
| **Department, n (%)**** | **0-11 years  (N = 430)** | **12-74 years  (N = 1192)** |  | **0-11 years  (N = 1170)** | **12-74 years  (N = 2310)** |  | **0-11 years  (N = 3270)** | **12-74 years  (N = 11580)** |  | **0-11 years  (N = 14550)** | **12-74 years  (N = 29523)** |
| Dermatology | 123 (28.6) | 503 (42.2) |  | 529 (45.2) | 1342 (58.1) |  | 1652 (50.5) | 6110 (52.8) |  | 8941 (61.5) | 20524 (69.5) |
| General internal medicine | 35 (8.1) | 357 (29.9) |  | 110 (9.4) | 470 (20.3) |  | 1157 (35.4) | 5120 (44.2) |  | 3679 (25.3) | 8643 (29.3) |
| Otolaryngology | 15 (3.5) | 101 (8.5) |  | 87 (7.4) | 150 (6.5) |  | 752 (23.0) | 2531 (21.9) |  | 1831 (12.6) | 2718 (9.2) |
| Pediatrics | 234 (54.4) | 35 (2.9) |  | 405 (34.6) | 52 (2.3) |  | 1738 (53.1) | 370 (3.2) |  | 5190 (35.7) | 845 (2.9) |
| Orthopedics | 3 (0.7) | 44 (3.7) |  | 15 (1.3) | 68 (2.9) |  | 51 (1.6) | 339 (2.9) |  | 218 (1.5) | 662 (2.2) |
| Urology | 3 (0.7) | 16 (1.3) |  | 14 (1.2) | 30 (1.3) |  | 62 (1.9) | 281 (2.4) |  | 294 (2.0) | 826 (2.8) |
| General surgery | 2 (0.5) | 52 (4.4) |  | 18 (1.5) | 91 (3.9) |  | 45 (1.4) | 279 (2.4) |  | 161 (1.1) | 517 (1.8) |
| Plastic surgery | 7 (1.6) | 6 (0.5) |  | 12 (1.0) | 22 (1.0) |  | 64 (2.0) | 254 (2.2) |  | 262 (1.8) | 692 (2.3) |
| Obstetrics and gynecology | 1 (0.2) | 47 (3.9) |  | 1 (0.1) | 116 (5.0) |  | 31 (0.9) | 207 (1.8) |  | 117 (0.8) | 654 (2.2) |
| Pulmonology | 0 (0.0) | 33 (2.8) |  | 0 (0.0) | 28 (1.2) |  | 11 (0.3) | 120 (1.0) |  | 20 (0.1) | 121 (0.4) |
| Gastroenterology | 1 (0.2) | 17 (1.4) |  | 2 (0.2) | 20 (0.9) |  | 6 (0.2) | 115 (1.0) |  | 44 (0.3) | 199 (0.7) |
| Ophthalmology | 1 (0.2) | 15 (1.3) |  | 2 (0.2) | 20 (0.9) |  | 17 (0.5) | 89 (0.8) |  | 56 (0.4) | 144 (0.5) |
| Department of Allergology | 13 (3.0) | 9 (0.8) |  | 23 (2.0) | 7 (0.3) |  | 36 (1.1) | 49 (0.4) |  | 103 (0.7) | 106 (0.4) |
| Neurosurgery | 1 (0.2) | 10 (0.8) |  | 2 (0.2) | 26 (1.1) |  | 6 (0.2) | 47 (0.4) |  | 13 (0.1) | 69 (0.2) |
| Cardiology | 0 (0.0) | 13 (1.1) |  | 0 (0.0) | 14 (0.6) |  | 4 (0.1) | 33 (0.3) |  | 14 (0.1) | 61 (0.2) |
| Rheumatology | 0 (0.0) | 15 (1.3) |  | 0 (0.0) | 11 (0.5) |  | 1 (0.0) | 30 (0.3) |  | 7 (0.0) | 20 (0.1) |
| Psychiatry | 0 (0.0) | 1 (0.1) |  | 0 (0.0) | 10 (0.4) |  | 6 (0.2) | 30 (0.3) |  | 23 (0.2) | 73 (0.2) |
| Neurology | 0 (0.0) | 16 (1.3) |  | 0 (0.0) | 14 (0.6) |  | 2 (0.1) | 23 (0.2) |  | 10 (0.1) | 21 (0.1) |
| Psychosomatic medicine | 0 (0.0) | 2 (0.2) |  | 0 (0.0) | 0 (0.0) |  | 0 (0.0) | 23 (0.2) |  | 4 (0.0) | 35 (0.1) |
| Other Surgeries | 0 (0.0) | 0 (0.0) |  | 0 (0.0) | 1 (0.0) |  | 5 (0.2) | 20 (0.2) |  | 10 (0.1) | 59 (0.2) |
| Other Departments | 0 (0.0) | 0 (0.0) |  | 0 (0.0) | 0 (0.0) |  | 0 (0.0) | 20 (0.2) |  | 0 (0.0) | 44 (0.1) |
| Department of Metabolism, Endocrinology and Diabetes | 0 (0.0) | 0 (0.0) |  | 0 (0.0) | 0 (0.0) |  | 0 (0.0) | 15 (0.1) |  | 2 (0.0) | 24 (0.1) |
| Other Internal Medicine | 0 (0.0) | 0 (0.0) |  | 0 (0.0) | 0 (0.0) |  | 8 (0.2) | 13 (0.1) |  | 14 (0.1) | 38 (0.1) |
| Respiratory surgery | 0 (0.0) | 3 (0.3) |  | 0 (0.0) | 4 (0.2) |  | 2 (0.1) | 12 (0.1) |  | 2 (0.0) | 6 (0.0) |
| Anesthesiology | 0 (0.0) | 2 (0.2) |  | 0 (0.0) | 3 (0.1) |  | 2 (0.1) | 11 (0.1) |  | 8 (0.1) | 10 (0.0) |
| Radiology | 0 (0.0) | 5 (0.4) |  | 0 (0.0) | 6 (0.3) |  | 1 (0.0) | 11 (0.1) |  | 2 (0.0) | 11 (0.0) |
| Emergency department | 10 (2.3) | 7 (0.6) |  | 11 (0.9) | 7 (0.3) |  | 12 (0.4) | 11 (0.1) |  | 15 (0.1) | 15 (0.1) |
| Dentistry | 0 (0.0) | 0 (0.0) |  | 0 (0.0) | 0 (0.0) |  | 4 (0.1) | 8 (0.1) |  | 14 (0.1) | 13 (0.0) |
| Cardiovascular surgery | 0 (0.0) | 1 (0.1) |  | 0 (0.0) | 6 (0.3) |  | 0 (0.0) | 7 (0.1) |  | 1 (0.0) | 15 (0.1) |
| Nephrology | 0 (0.0) | 0 (0.0) |  | 0 (0.0) | 0 (0.0) |  | 0 (0.0) | 4 (0.0) |  | 1 (0.0) | 11 (0.0) |
| Neuropsychiatry | 1 (0.2) | 0 (0.0) |  | 0 (0.0) | 1 (0.0) |  | 2 (0.1) | 3 (0.0) |  | 1 (0.0) | 4 (0.0) |
| Department of Rehabilitation | 0 (0.0) | 2 (0.2) |  | 0 (0.0) | 3 (0.1) |  | 1 (0.0) | 3 (0.0) |  | 8 (0.1) | 5 (0.0) |

*Missing information on medical departments was imputed using the primary medical department listed for each institution.

**Patients with prescriptions from multiple medical institutes during the follow-up period were counted multiple times.

**Table S13.** Breakdown of departments (practitioner and hospital doctor) prescribing OCS/TCS in CSU Cohort during the follow-up period

|  | **OCS**  **(without imputation*)** | |  | **TCS**  **(without imputation*)** | |  | **OCS**  **(with imputation*)** | |  | **TCS**  **(with imputation*)** | |
| --- | --- | --- | --- | --- | --- | --- | --- | --- | --- | --- | --- |
| **Department, n (%)**** | **0-11 years  (N = 430)** | **12-74 years  (N = 1192)** |  | **0-11 years  (N = 1177)** | **12-74 years  (N = 2310)** |  | **0-11 years  (N = 3270)** | **12-74 years  (N = 11580)** |  | **0-11 years  (N = 14550)** | **12-74 years  (N = 29523)** |
| All departments |  |  |  |  |  |  |  |  |  |  |  |
| Practitioner | 179 (41.6) | 481 (40.4) |  | 691 (58.7) | 1134 (49.1) |  | 3121 (95.4) | 10660 (92.1) |  | 13908 (95.6) | 27402 (92.8) |
| Hospital doctor | 263 (61.2) | 732 (61.4) |  | 509 (43.2) | 1210 (52.4) |  | 666 (20.4) | 2088 (18.0) |  | 1813 (12.5) | 4097 (13.9) |
| Dermatology | 123 | 503 |  | 529 | 1342 |  | 1652 | 6110 |  | 8941 | 20524 |
| Practitioner | 105 (85.4) | 239 (47.5) |  | 449 (84.9) | 756 (56.3) |  | 1648 (99.8) | 5941 (97.2) |  | 8894 (99.5) | 20089 (97.9) |
| Hospital doctor | 19 (15.4) | 268 (53.3) |  | 83 (15.7) | 594 (44.3) |  | 19 (1.2) | 270 (4.4) |  | 84 (0.9) | 598 (2.9) |
| General internal medicine | 35 | 357 |  | 110 | 470 |  | 1157 | 5120 |  | 3679 | 8643 |
| Practitioner | 35 (100.0) | 162 (45.4) |  | 104 (94.5) | 237 (50.4) |  | 791 (68.4) | 3822 (74.6) |  | 2573 (69.9) | 6029 (69.8) |
| Hospital doctor | 0 (0.0) | 197 (55.2) |  | 6 (5.5) | 235 (50.0) |  | 454 (39.2) | 1583 (30.9) |  | 1290 (35.1) | 3014 (34.9) |
| Otolaryngology | 15 | 101 |  | 87 | 150 |  | 752 | 2531 |  | 1831 | 2718 |
| Practitioner | 11 (73.3) | 44 (43.6) |  | 68 (78.2) | 64 (42.7) |  | 748 (99.5) | 2469 (97.6) |  | 1811 (98.9) | 2631 (96.8) |
| Hospital doctor | 4 (26.7) | 57 (56.4) |  | 19 (21.8) | 86 (57.3) |  | 4 (0.5) | 66 (2.6) |  | 21 (1.1) | 95 (3.5) |
| Pediatrics | 234 | 35 |  | 405 | 52 |  | 1738 | 370 |  | 5190 | 845 |
| Practitioner | 24 (10.3) | 12 (34.3) |  | 58 (14.3) | 12 (23.1) |  | 1588 (91.4) | 339 (91.6) |  | 4904 (94.5) | 788 (93.3) |
| Hospital doctor | 211 (90.2) | 23 (65.7) |  | 348 (85.9) | 40 (76.9) |  | 248 (14.3) | 31 (8.4) |  | 429 (8.3) | 60 (7.1) |
| Orthopedics | 3 | 44 |  | 15 | 68 |  | 51 | 339 |  | 218 | 662 |
| Practitioner | 1 (33.3) | 6 (13.6) |  | 8 (53.3) | 15 (22.1) |  | 39 (76.5) | 266 (78.5) |  | 186 (85.3) | 509 (76.9) |
| Hospital doctor | 2 (66.7) | 38 (86.4) |  | 7 (46.7) | 53 (77.9) |  | 13 (25.5) | 76 (22.4) |  | 35 (16.1) | 157 (23.7) |
| Urology | 3 | 16 |  | 14 | 30 |  | 62 | 281 |  | 294 | 826 |
| Practitioner | 2 (66.7) | 1 (6.3) |  | 6 (42.9) | 4 (13.3) |  | 61 (98.4) | 264 (94.0) |  | 285 (96.9) | 794 (96.1) |
| Hospital doctor | 1 (33.3) | 15 (93.8) |  | 8 (57.1) | 26 (86.7) |  | 1 (1.6) | 17 (6.0) |  | 9 (3.1) | 32 (3.9) |
| General surgery | 2 | 52 |  | 18 | 91 |  | 45 | 279 |  | 161 | 517 |
| Practitioner | 0 (0.0) | 4 (7.7) |  | 7 (38.9) | 11 (12.1) |  | 41 (91.1) | 205 (73.5) |  | 141 (87.6) | 392 (75.8) |
| Hospital doctor | 2 (100.0) | 48 (92.3) |  | 11 (61.1) | 80 (87.9) |  | 4 (8.9) | 74 (26.5) |  | 20 (12.4) | 125 (24.2) |
| Plastic surgery | 7 | 6 |  | 12 | 22 |  | 64 | 254 |  | 262 | 692 |
| Practitioner | 0 (0.0) | 2 (33.3) |  | 1 (8.3) | 4 (18.2) |  | 57 (89.1) | 247 (97.2) |  | 250 (95.4) | 671 (97.0) |
| Hospital doctor | 7 (100.0) | 4 (66.7) |  | 11 (91.7) | 18 (81.8) |  | 7 (10.9) | 7 (2.8) |  | 12 (4.6) | 21 (3.0) |
| Obstetrics and gynecology | 1 | 47 |  | 1 | 116 |  | 31 | 207 |  | 117 | 654 |
| Practitioner | 1 (100.0) | 14 (29.8) |  | 1 (100.0) | 38 (32.8) |  | 18 (58.1) | 165 (79.7) |  | 75 (64.1) | 537 (82.1) |
| Hospital doctor | 0 (0.0) | 33 (70.2) |  | 0 (0.0) | 78 (67.2) |  | 13 (41.9) | 43 (20.8) |  | 42 (35.9) | 122 (18.7) |
| Pulmonology | 0 | 33 |  | 0 | 28 |  | 11 | 120 |  | 20 | 121 |
| Practitioner | – | 1 (3.0) |  | – | 0 (0.0) |  | 5 (45.5) | 58 (48.3) |  | 7 (35.0) | 43 (35.5) |
| Hospital doctor | – | 32 (97.0) |  | – | 28 (100.0) |  | 6 (54.5) | 62 (51.7) |  | 13 (65.0) | 78 (64.5) |
| Gastroenterology | 1 | 17 |  | 2 | 20 |  | 6 | 115 |  | 44 | 199 |
| Practitioner | 1 (100.0) | 0 (0.0) |  | 1 (50.0) | 1 (5.0) |  | 5 (83.3) | 85 (73.9) |  | 37 (84.1) | 157 (78.9) |
| Hospital doctor | 0 (0.0) | 17 (100.0) |  | 1 (50.0) | 19 (95.0) |  | 1 (16.7) | 30 (26.1) |  | 7 (15.9) | 43 (21.6) |
| Ophthalmology | 1 | 15 |  | 2 | 20 |  | 17 | 89 |  | 56 | 144 |
| Practitioner | 0 (0.0) | 3 (20.0) |  | 0 (0.0) | 8 (40.0) |  | 16 (94.1) | 75 (84.3) |  | 54 (96.4) | 130 (90.3) |
| Hospital doctor | 1 (100.0) | 12 (80.0) |  | 2 (100.0) | 12 (60.0) |  | 1 (5.9) | 14 (15.7) |  | 2 (3.6) | 14 (9.7) |
| Department of Allergology | 13 | 9 |  | 23 | 7 |  | 36 | 49 |  | 103 | 106 |
| Practitioner | 0 (0.0) | 0 (0.0) |  | 0 (0.0) | 0 (0.0) |  | 23 (63.9) | 40 (81.6) |  | 80 (77.7) | 99 (93.4) |
| Hospital doctor | 13 (100.0) | 9 (100.0) |  | 23 (100.0) | 7 (100.0) |  | 13 (36.1) | 9 (18.4) |  | 23 (22.3) | 7 (6.6) |
| Neurosurgery | 1 | 10 |  | 2 | 26 |  | 6 | 47 |  | 13 | 69 |
| Practitioner | 0 (0.0) | 0 (0.0) |  | 0 (0.0) | 1 (3.8) |  | 4 (66.7) | 30 (63.8) |  | 10 (76.9) | 36 (52.2) |
| Hospital doctor | 1 (100.0) | 10 (100.0) |  | 2 (100.0) | 25 (96.2) |  | 2 (33.3) | 17 (36.2) |  | 3 (23.1) | 33 (47.8) |
| Cardiology | 0 | 13 |  | 0 | 14 |  | 4 | 33 |  | 14 | 61 |
| Practitioner | – | 1 (7.7) |  | – | 1 (7.1) |  | 2 (50.0) | 19 (57.6) |  | 11 (78.6) | 38 (62.3) |
| Hospital doctor | – | 12 (92.3) |  | – | 13 (92.9) |  | 2 (50.0) | 14 (42.4) |  | 3 (21.4) | 23 (37.7) |
| Rheumatology | 0 | 15 |  | 0 | 11 |  | 1 | 30 |  | 7 | 20 |
| Practitioner | – | 2 (13.3) |  | – | 1 (9.1) |  | 1 (100.0) | 14 (46.7) |  | 5 (71.4) | 8 (40.0) |
| Hospital doctor | – | 13 (86.7) |  | – | 10 (90.9) |  | 0 (0.0) | 16 (53.3) |  | 2 (28.6) | 12 (60.0) |
| Psychiatry | 0 | 1 |  | 0 | 10 |  | 6 | 30 |  | 23 | 73 |
| Practitioner | – | 0 (0.0) |  | – | 0 (0.0) |  | 1 (16.7) | 18 (60.0) |  | 8 (34.8) | 35 (47.9) |
| Hospital doctor | – | 1 (100.0) |  | – | 10 (0.0) |  | 5 (83.3) | 12 (40.0) |  | 15 (65.2) | 38 (52.1) |
| Neurology | 0 | 16 |  | 0 | 14 |  | 2 | 23 |  | 10 | 21 |
| Practitioner | – | 0 (0.0) |  | 0 (0.0) | 0 (0.0) |  | 1 (50.0) | 6 (26.1) |  | 1 (10.0) | 6 (28.6) |
| Hospital doctor | – | 16 (100.0) |  | 0 (0.0) | 14 (100.0) |  | 1 (50.0) | 17 (73.9) |  | 9 (90.0) | 15 (71.4) |
| Psychosomatic medicine | 0 | 2 |  | 0 | 1 (50.0) |  | 0 | 23 |  | 4 | 35 |
| Practitioner | – | 1 (50.0) |  | – | 1 (50.0) |  | – | 22 (95.7) |  | 4 (100.0) | 35 (100.0) |
| Hospital doctor | – | 1 (50.0) |  | – | 1 (50.0) |  | – | 1 (4.3) |  | 0 (0.0) | 0 (0.0) |
| Miscellaneous Surgery | 0 | 0 |  | 0 | 1 |  | 5 | 20 |  | 10 | 59 |
| Practitioner | – | – |  | – | 0 (0.0) |  | 5 (100.0) | 16 (80.0) |  | 10 (100.0) | 50 (84.7) |
| Hospital doctor | – | – |  | – | 1 (100.0) |  | 0 (0.0) | 4 (20.0) |  | 0 (0.0) | 9 (15.3) |
| Other Families | 0 | 0 |  | 0 | 0 |  | 0 | 20 |  | 0 | 44 |
| Practitioner | – | – |  | – | – |  | – | 14 (70.0) |  | – | 36 (81.8) |
| Hospital doctor | – | – |  | – | – |  | – | 6 (30.0) |  | – | 8 (18.2) |
| Department of Metabolism, Endocrinology and Diabetes | 0 | 0 |  | 0 | 0 |  | 0 | 15 |  | 2 | 24 |
| Practitioner | – | – |  | – | – |  | – | 5 (33.3) |  | 0 (0.0) | 10 (41.7) |
| Hospital doctor | – | – |  | – | – |  | – | 10 (66.7) |  | 2 (100.0) | 14 (58.3) |
| Other Internal Medicine | 0 | 0 |  | 0 | 0 |  | 8 | 13 |  | 14 | 38 |
| Practitioner | – | – |  | – | – |  | 8 (100.0) | 9 (69.2) |  | 12 (85.7) | 30 (78.9) |
| Hospital doctor | – | – |  | – | – |  | 0 (0.0) | 4 (30.8) |  | 2 (14.3) | 8 (21.1) |
| Respiratory surgery | 0 | 3 |  | 0 | 4 |  | 2 | 12 |  | 2 | 6 |
| Practitioner | – | 0 (0.0) |  | – | 0 (0.0) |  | 2 (100.0) | 9 (75.0) |  | 2 (100.0) | 2 (33.3) |
| Hospital doctor | – | 3 (100.0) |  | – | 4 (100.0) |  | 0 (0.0) | 3 (25.0) |  | 0 (0.0) | 4 (66.7) |
| Anesthesiology | 0 | 2 |  | 0 | 3 |  | 2 | 11 |  | 8 | 10 |
| Practitioner | – | 0 (0.0) |  | – | 0 (0.0) |  | 2 (100.0) | 9 (81.8) |  | 8 (100.0) | 7 (70.0) |
| Hospital doctor | – | 2 (100.0) |  | – | 3 (100.0) |  | 0 (0.0) | 2 (18.2) |  | 0 (0.0) | 3 (30.0) |
| Radiology | 0 | 5 |  | 0 | 6 |  | 1 | 11 |  | 2 | 11 |
| Practitioner | – | 0 (0.0) |  | – | 0 (0.0) |  | 1 (100.0) | 5 (45.5) |  | 2 (100.0) | 4 (36.4) |
| Hospital doctor | – | 5 (100.0) |  | – | 6 (100.0) |  | 0 (0.0) | 6 (54.5) |  | 0 (0.0) | 7 (63.6) |
| Emergency department | 10 | 7 |  | 11 | 7 |  | 12 | 11 |  | 15 | 15 |
| Practitioner | 0 (0.0) | 0 (0.0) |  | 0 (0.0) | 0 (0.0) |  | 0 (0.0) | 0 (0.0) |  | 0 (0.0) | 0 (0.0) |
| Hospital doctor | 10 (100.0) | 7 (100.0) |  | 11 (100.0) | 7 (100.0) |  | 12 (100.0) | 11 (100.0) |  | 15 (100.0) | 15 (100.0) |
| Dentistry | 0 | 0 |  | 0 | 0 |  | 4 | 8 |  | 14 | 13 |
| Practitioner | – | – |  | – | – |  | 4 (100.0) | 7 (87.5) |  | 14 (100.0) | 11 (84.6) |
| Hospital doctor | – | – |  | – | – |  | 0 (0.0) | 1 (12.5) |  | 0 (0.0) | 2 (15.4) |
| Cardiovascular surgery | 0 | 1 |  | 0 | 6 |  | 0 | 7 |  | 1 | 15 |
| Practitioner | – | 0 (0.0) |  | – | 0 (0.0) |  | – | 0 (0.0) |  | 0 (0.0) | 1 (6.7) |
| Hospital doctor | – | 1 (100.0) |  | – | 6 (100.0) |  | – | 7 (100.0) |  | 1 (100.0) | 14 (93.3) |
| Nephrology | 0 | 0 |  | 0 | 0 |  | 0 | 4 |  | 1 | 11 |
| Practitioner | – | – |  | – | – |  | – | 2 (50.0) |  | 1 (100.0) | 9 (81.8) |
| Hospital doctor | – | – |  | – | – |  | – | 2 (50.0) |  | 0 (0.0) | 2 (18.2) |
| Neurology | 1 | 0 |  | 0 | 1 |  | 2 | 3 |  | 1 | 4 |
| Practitioner | 0 (0.0) | – |  | – | 0 (0.0) |  | 1 (50.0) | 3 (100.0) |  | 1 (100.0) | 3 (75.0) |
| Hospital doctor | 1 (100.0) | – |  | – | 1 (100.0) |  | 1 (50.0) | 0 (0.0) |  | 0 (0.0) | 1 (25.0) |
| Department of Rehabilitation | 0 | 2 |  | 0 | 3 |  | 1 | 3 |  | 8 | 5 |
| Practitioner | – | 0 (0.0) |  | – | 0 (0.0) |  | 0 (0.0) | 1 (33.3) |  | 0 (0.0) | 1 (20.0) |
| Hospital doctor | – | 2 (100.0) |  | – | 3 (100.0) |  | 1 (100.0) | 2 (66.7) |  | 8 (100.0) | 4 (80.0) |

*Missing information on medical departments was imputed using the primary medical department listed for each institution.

**Patients with prescriptions from multiple medical institutes during the follow-up period were counted multiple times.

**Figure S1.** Sankey diagram showing number (percentage) of adolescent/adult (12-74 years) CSU patients migrating at each transition step (n=57,045)


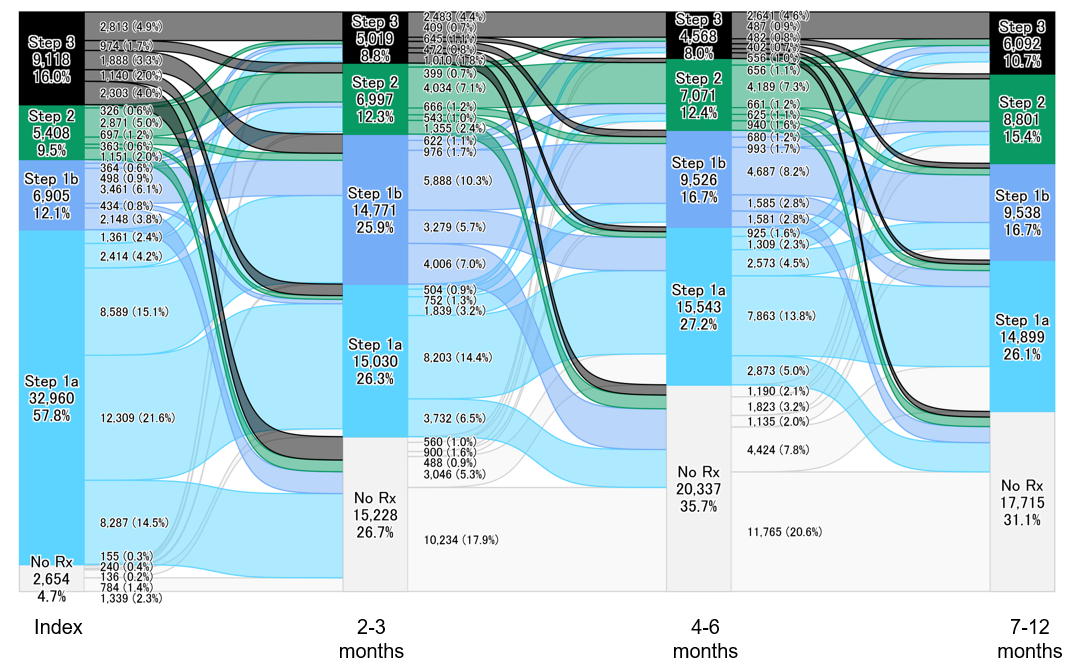

Supplement: Supplementary file 1 — Data S1: jde17943‐sup‐0001‐DataS1.docx. [file JDE-52-1775-s001.docx]
